# Allosteric Covalent Inhibitors of the STAT3 Transcription Factor from Virtual Screening

**DOI:** 10.1021/acsmedchemlett.4c00622

**Published:** 2025-05-06

**Authors:** Tibor Viktor Szalai, Vincenzo di Lorenzo, Nikolett Péczka, Levente M. Mihalovits, László Petri, Qirat F. Ashraf, Elvin D. de Araujo, Viktor Honti, Dávid Bajusz, György M. Keserű

**Affiliations:** † Medicinal Chemistry Research Group, 280964 HUN-REN Research Centre for Natural Sciences, Magyar tudósok krt. 2, 1117 Budapest, Hungary; ‡ National Drug Research and Development Laboratory, HUN-REN Research Centre for Natural Sciences, Magyar tudósok krt. 2, 1117 Budapest, Hungary; ◧ Department of Inorganic and Analytical Chemistry, Faculty of Chemical Technology and Biotechnology, Budapest University of Technology and Economics, Műegyetem rkp. 3, H-1111 Budapest, Hungary; ∥ Department of Organic Chemistry and Technology, Faculty of Chemical Technology and Biotechnology, Budapest University of Technology and Economics, Műegyetem rkp. 3, H-1111 Budapest, Hungary; ⊥ Department of Chemical & Physical Sciences, 71637 University of Toronto Mississauga, Mississauga, ON L5L 1C6, Canada; # Centre for Medicinal Chemistry, University of Toronto at Mississauga, Mississauga, ON L5L 1C6, Canada; ○ HUN-REN Biological Research Centre, Institute of Genetics, Drosophila Blood Cell Differentiation Group, 62. Temesvári krt., 6726 Szeged, Hungary

**Keywords:** Virtual screening, covalent inhibitor, allosteric site, transcription factor, coiled-coil domain

## Abstract

The STAT family of transcription factors are important signaling hubs, with several of them, particularly STAT3, being emerging oncotargets already investigated in clinical trials. The modular structure of STAT3 nominates several of its protein domains as possible drug targets, but their exploitation with potential small-molecule inhibitors has been unevenly distributed so far, with past efforts highly favoring the conserved SH2 domain. Here, we have targeted a sparsely studied binding site at the junction of the coiled-coil and DNA-binding domains and discovered several new lead-like covalent inhibitors by virtual screening. The most favorable hit compound has been explored via structure-guided hit expansion and optimized into a low micromolar inhibitor. This compound can serve as a chemical biology tool against this site in future exploratory studies or form the basis of a more advanced stage of lead optimization.

## Graphical abstract

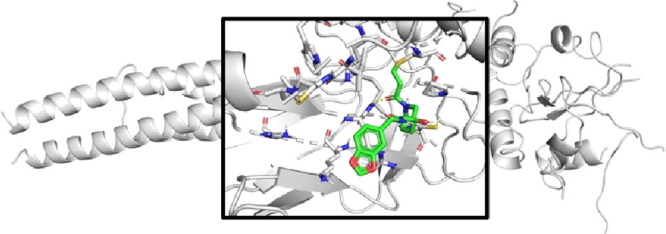

Signal transducers and activators of transcription (STATs) are a small family of important oncotargets that act as hubs in intracellular signaling and, upon activation, directly mediate DNA transcription in the nucleus.^1–3^ Their importance is underscored by their involvement in a wide range of leukemiae,^4–7^ as well as solid cancers.^8,9^ STATs exhibit a modular architecture with six distinct domains that were described, characterized, and targeted to various extents. Chiefly, the most attention was dedicated to the SH2 domain that is responsible for the formation of the active STAT dimer (capable of binding DNA^10^) through *vis-à-vis* docking of a phosphorylated tyrosine residue on the C-terminal segment of the partner monomer, within a small cavity that contains a conserved arginine residue.^11,12^ Most of the small-molecule STAT inhibitors described so far target the SH2 domain, with STAT3 and STAT5B being the most important targets of this family.^13^ A key disadvantage of this site though is its strong preference for phosphate or other heavily charged phosphate-mimetic groups, which has a negative impact on bioavailability and ADME properties in general. Comparatively less effort was dedicated to developing directly competitive DNA-binding domain (DBD) inhibitors,^14,15^ although a proof-of-concept study on flavopiridol as a DBD inhibitor was published as early as 2006.^16^ Beyond dimers, a higher level of the structural organization of STATs is the formation of tetramers via linkage of the N-terminal domains of the active dimers—a process that was shown to contribute to a finer control of gene expression^13^ and in turn contribute to natural killer cell homeostasis^17^ and leukemogenesis.^18^ Early examples for targeting the N-terminal domain are an ultrahigh throughput virtual screening effort that resulted in new STAT3 inhibitors,^19^ and our recent work that revealed potent hits against the STAT5B N-terminal domain from a photoaffinity-tagged fragment library.^200^ A further proof-of-concept effort, and direct conceptual basis of the present study, is the work of the Surh lab on prostaglandin analogues that were shown to covalently target the C259 residue of the coiled-coil domain (CCD), close to its interface with the DBD.^20,21^ The small molecules were validated *in vitro* as promising suppressors of breast cancer cell growth. With that, targeting the described allosteric site comprises a promising orthogonal strategy to targeting the SH2 domain; key advantages include the elimination of a charged group, and possibly more easily achievable selectivity against six other STATs (vs 120 other SH2 domains).^22^

Covalent modification is a main protein targeting mechanism, consisting of two consecutive steps.^23^ These involve the formation of secondary interactions between the ligand and the protein environment of the binding pocket (noncovalent step) and the formation of the covalent bond between the targeted nucleophilic side chain and the electrophilic warhead of the ligand (covalent step). The design of targeted covalent inhibitors (TCIs) has become a cornerstone of drug discovery efforts since its reemergence around the turn of the millennia, owing to their advantages over their noncovalent counterparts.^24^ These include prolonged target engagement, higher therapeutic index, distinct pharmacodynamic profile, exceptional potency, and lower dosage requirement.^25^ With proper tuning of the warhead reactivity, along with the optimization of secondary protein–ligand interactions, previous concerns regarding off-target toxicity and selectivity issues of covalent inhibition can be alleviated. Although various nucleophilic side chains were labeled previously,^26–29^ cysteine targeting inhibitors still provide the highest contribution to TCIs owing to cysteine’s pronounced nucleophilic character and its low occurrence in the human proteome,^30^ the latter minimizing off-target reactivity of such compounds. Acrylamides are among the most prevalent electrophiles used as warhead during the design of cysteine targeting inhibitors; publications regarding this specific group of molecules are numerous.^31,32^ Over the past 10 years the development of covalent inhibitors has been in the forefront of medicinal chemistry, with over 55 marketed covalent drugs as of late.^23,33^ Covalent inhibitors are used in major indications among CNS, cardiovascular, and gastrointestinal disorders along with infections; however, the main therapeutic area is oncology. About 28% of marketed covalent inhibitors are applied in cancer-related therapies;^23^ hence, the discovery of novel STAT3 inhibitors is highly relevant for the pharmaceutical industry.

Here, we set out to discover a more drug-like chemical starting point against the allosteric binding site of the coiled-coil domain of STAT3, utilizing the C259 anchoring residue for covalent attachment. Following successful hit generation via virtual screening and validation of the binding site by mass spectrometry, a low micromolar inhibitor was achieved by a combinatorial hit follow-up.

## Virtual Screening and Hit Follow-up

To discover chemical starting points for an allosteric covalent inhibitor of STAT3, we have performed structure-based virtual screening of the Enamine Acrylamide library [2022.01.06. version, access date: 2022.10.10] (the largest commercial source of acrylamides) against the C259 residue of STAT3, with covalent docking (Figure 1). Ten compounds were purchased from Enamine after cherry-picking from the virtual screening results, based on binding site complementarity and physical availability. Primary hit verification was conducted with an *in vitro* fluorescence polarization assay. The active acrylamides and their IC_50_ values are shown in Figure 2a.

**Figure 1:**
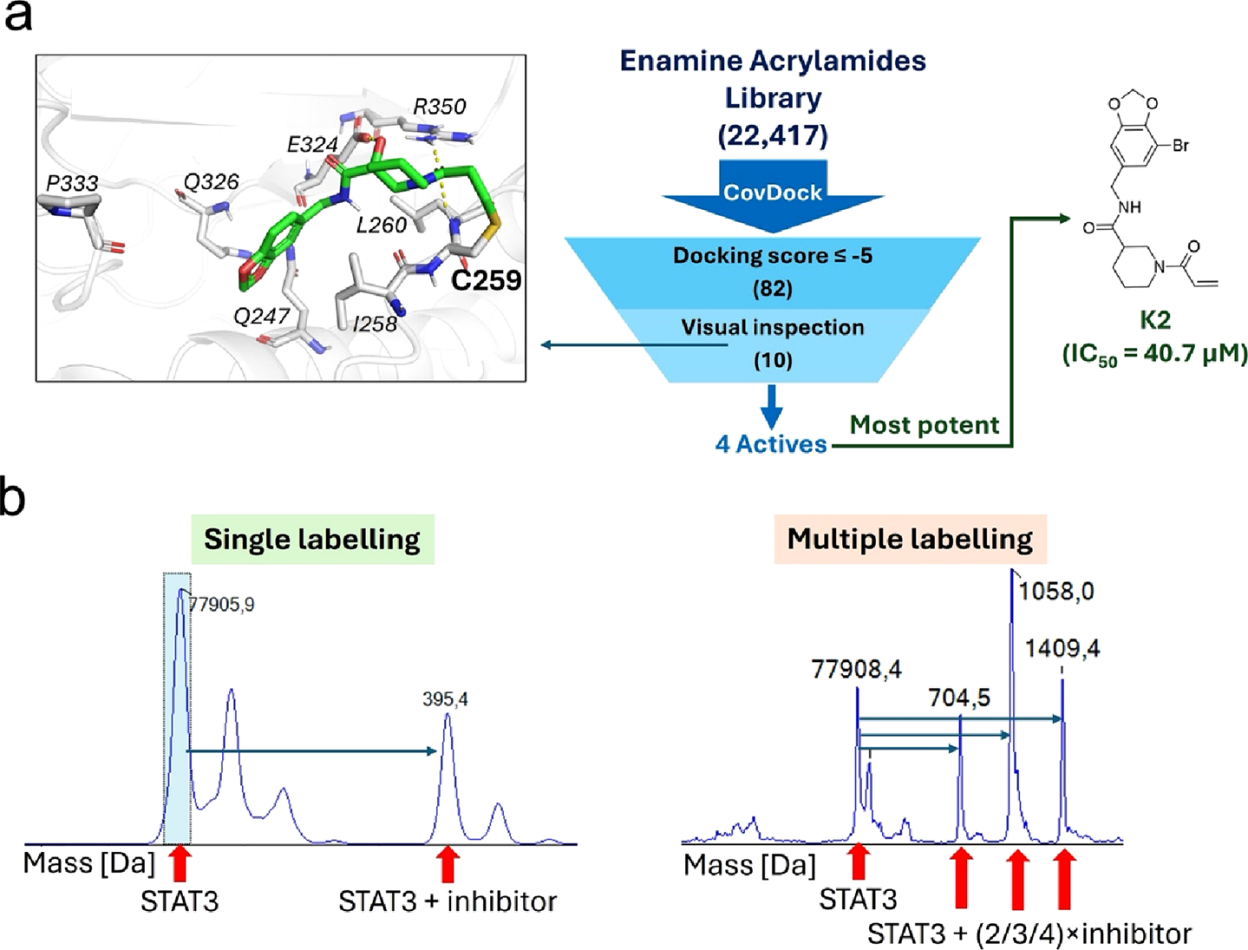
Overview of the virtual screening workflow and hit follow-up. (a) Overview of the covalent docking virtual screening workflow, including representation of the key residues inside the binding site and highlighting the C259 residue. Remaining molecules after each step are included in parentheses. (b) Schematic representation of the expected mass peaks and shifts from the MS results for single and multiple labeling. The values assigned to the peaks for other than the peak corresponding to the unlabeled protein represents mass shift(s) resulting from successful labeling. Expected mass shifts are equal to the molecular weight of the inhibitor for single labeling and whole number multiples of the molecular weight of the inhibitor for multiple labeling.

**Figure 2:**
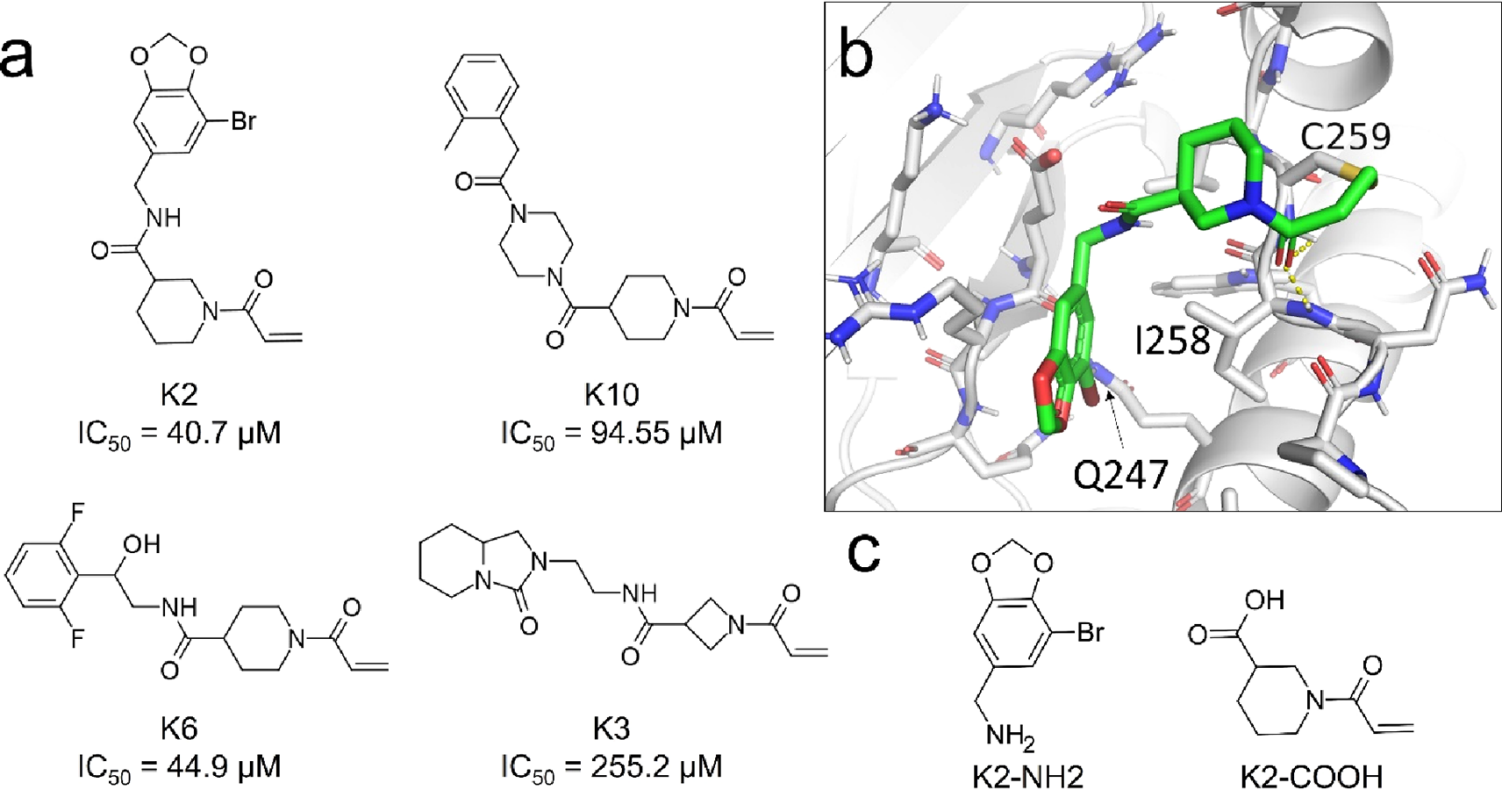
(a) Confirmed hits from the Enamine Acrylamides library, with their respective IC_50_ values. (b) Modeled binding mode of K2 in the targeted binding site of STAT3. K2 interacts with the Q247 residue and the protein backbone at the I258 and C259 residues. Hydrogen bonds are highlighted with yellow dashed lines. (c) Structure of the building blocks of K2.

The most potent inhibitor of the confirmed hits was K2, which was used as the basis for hit follow-up. K2 was split along its amide bond, resulting in building blocks K2-NH2 and K2-COOH (Figure 2c). Compounds similar to K2-NH2 and K2-COOH were retrieved from the databases of online vendors, and a total of 21 new amine building blocks and 14 new carboxylic acid building blocks were selected (Figures S1 and S2). These compounds (along with K2-NH2 and K2-COOH themselves) were then combinatorially reacted to obtain a total of 330 compounds (one of them being K2 itself). The 330 compounds (K2-analogues) were covalently docked to the C259 residue, and the results were ranked based on the docking scores (DS).

The building block analysis of the top 100 compounds based on DS (Figure S3) has shown that amines A14 and A15 and the carboxylic acid C2 were the most abundant building blocks among the top 100 compounds (Figure 3a). Based on the predicted binding modes and the availability of the building blocks, A14 and C2 were chosen to synthesize A14C2 (Figure 3b). It is interesting to note that C2 is a close analogue of K2-COOH, with an additional hydroxyl group on the piperidine ring. Examining its predicted binding mode, this hydroxyl group can establish a hydrogen bond with the E324 residue of STAT3, which explains the consistently better docking scores of compounds with C2 as their carboxylic acid building block instead of K2-COOH (Figure 3c).

**Figure 3:**
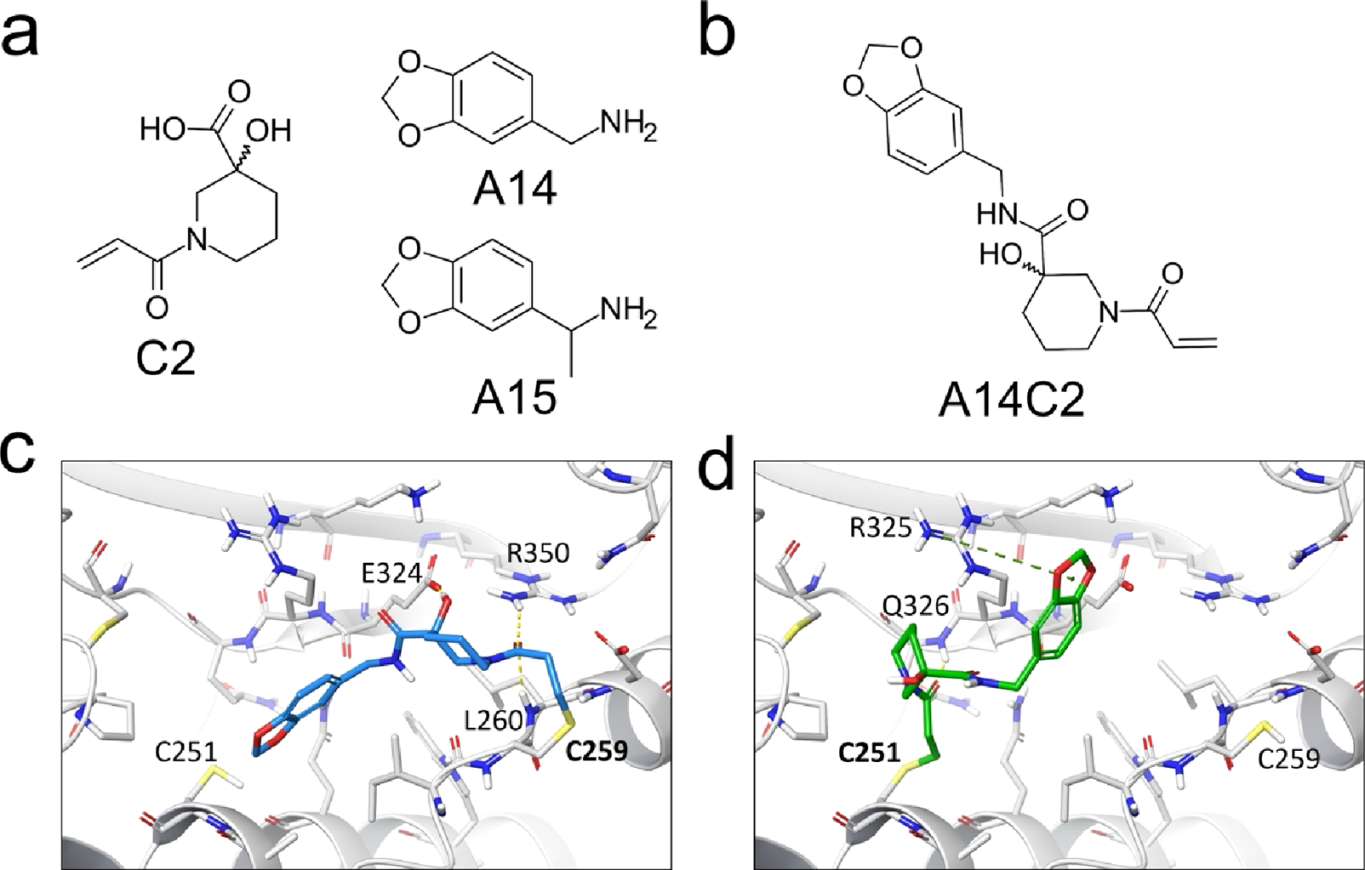
(a) Most abundant building blocks out of the top 100 K2-analogues. (b) Structure of A14C2. (c) Predicted binding mode of A14C2 (**6b**) against the C259 residue in the allosteric binding site of STAT3. (d) Predicted binding mode of A14C2 (**6b**) in the same site against its experimentally confirmed binding partner, C251. Secondary interactions are highlighted with dashed lines (H-bond: yellow; cation-pi: green).

To improve the activity of hit K2, two alternative strategies were tried. In addition to the optimization of noncovalent interactions based on the building block analysis (**6a,b**), the other route was the application of a more reactive warhead, namely, vinyl-sulfone (**8**). To explore the role of covalent bond formation, we also designed a noncovalent analogue with similar size (**10**). For the synthesis of the designed compounds, A14 (**1**) was reacted with the appropriate 1-Boc-nipecotic acid (**2a,b**) via a standard coupling reaction, and then the Boc protecting group was removed. As the final steps, **4a,b** was acylated, resulting in compounds **6a,b**, **8**, and **10a,b** (Scheme 1).

**Scheme 1:**
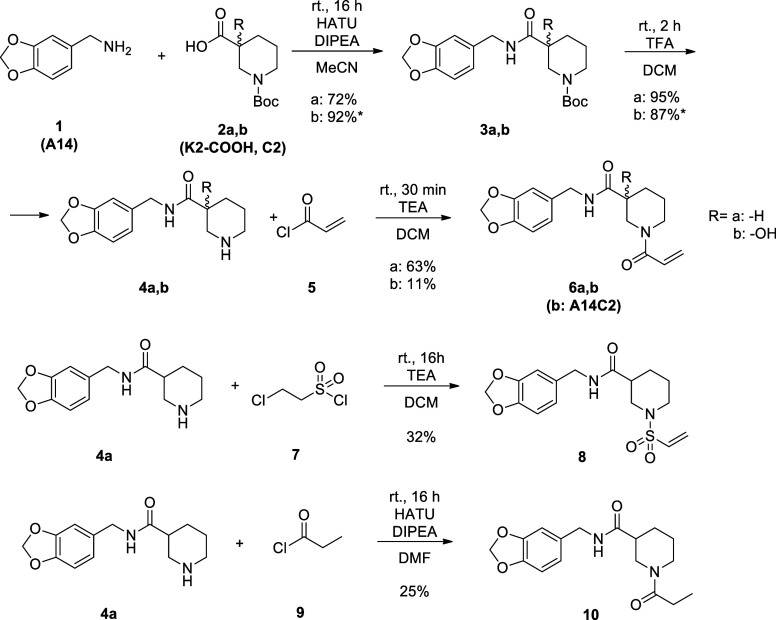
Synthesis of the K2 Analogues^a^

a Yields marked with * are crude yields.

The activity of the compounds was measured in a fluorescence polarization assay, and covalent bond formation was confirmed via intact MS measurements. Compound **6a** showed similar activity in the FP assay with a 50.4 ± 39.41 μM IC_50_ (mean ± 95% confidence interval), as the original hit K2 (IC_50_ = 40.7 ± 16.7 μM) and labeled the STAT3 protein at the targeted C259 residue (see Supplementary Figures S4 and S5). Its vinyl-sulfone analogue, compound **8**, showed a somewhat improved activity (IC_50_ = 33.4 ± 5.0 μM) compared to the acrylamides K2 and **6a**; however, it proved to be less selective, as it labeled the protein at multiple residues (see Supplementary Figure S4). This reduced selectivity can be attributed to the inherently higher electrophilic reactivity of **8** due to its stronger Michael acceptor properties, together with its modest noncovalent affinity that is insufficient to restrict reactivity to unwanted binding sites. The application of building block C2 in compound **6b** improved the activity more than 10-fold (IC_50_ = 2.91 ± 0.66 μM), while retaining selectivity (single labeling), nominating it as the advanced hit of this series. Interestingly, compound **6b** was found to label the C251 residue in the peptide mapping experiment, which is located at the opposite side of the binding site relative to C259, resulting in a flipped binding mode of compound **6b** (Figure 3d). This finding highlights the necessity of accounting for both targetable cysteines when targeting this site and suggests that the binding mode of the ligand can be fine-tuned by the strategic placement of substituents. The noncovalent molecules (**10a** and **10b**) showed reduced activity in the FP assay with a 140.7 ± 53.4 μM and 86.7 ± 49.9 μM IC_50_ values, respectively. These results point at a more dominant contribution of the covalent warhead for molecule **6b** (than **6a**) in achieving affinity toward STAT3, which might be a result of the different binding mode and site of labeling (C251 vs C259).

Structure-based virtual library screening of commercially available acrylamides has successfully identified several initial hits against the sparsely studied allosteric site at the interface of the coiled-coil and DNA-binding domains of STAT3. The hits were validated with a fluorescence polarization assay, and mass spectrometry was used to confirm the covalent mode of action, as well as the binding site. The most active virtual screening hit **K2** was selected as the basis of hit expansion with a combinatorial strategy, where a small virtual library was compiled by joining commercially available building blocks and a second round of virtual screening was performed to select and synthesize the most promising analogues. This effort has resulted in a low micromolar, allosteric covalent inhibitor of STAT3 as a promising tool compound against this important oncotarget. While a detailed in vivo characterization was out of the scope of the current study, recent studies suggest antiproliferative and proapoptotic activities of compounds targeting this binding site, particularly in H-Ras-transformed human mammary epithelial cells (MCF10A-Ras), via the inhibition of the phosphorylation, dimerization, nuclear translocation, and transcriptional activity of STAT3. Further efforts to achieve a better understanding of the in vivo effects (including potential unwanted side-effects) of these molecules should entail a detailed characterization of potential orthogonal mechanisms of action via suitable chemoproteomics measurements.

Finally, to complement the early work we reported here, we note a few future directions that can shape this effort. More sophisticated computational design tools like QM/MM molecular dynamics simulations can be deployed to gain a more detailed understanding of the mechanism of action for the reported series of TCIs, which can, in turn, guide prospective design. Also, although we used the largest library of readily available acrylamides in this work, the accessible chemical space is quickly growing, and on-demand virtual libraries could provide an even more comprehensive search space for similar efforts. This of course results in an increased computational demand, for which AI-assisted virtual screening workflows will hopefully provide an economic alternative in the near future (at the time of writing, these are available for screening noncovalent compounds). Likewise, for the prediction of pharmacokinetic properties by advanced cheminformatics tools, the scarcity of training data for targeted covalent inhibitors will likely stop being a limiting factor in the future, unlocking this invaluable set of computational tools for this compound class.

## Experimental Procedures

### Virtual Screening and Hit Follow-Up

The Acrylamides library from Enamine (2022.01.06. version), containing 22,417 acrylamides, was prepared using LigPrep^35^ at a pH of 7.4. The prepared library was then covalently docked using CovDock^36^ with thorough Pose Prediction docking mode with Michael Addition reaction type to the C259 residue on STAT3 (PDB ID: 6QHD).^37^ No additional constraint has been set. Results were ranked based on docking score (DS), and compounds with DS ≤ −5 were visually inspected, starting with the compound possessing the most negative DS value. A compound was selected if it did not have a significant part of it in a solvent-exposed region (i.e., without any contact with the protein) AND it had at least one advantageous secondary interaction with the protein (in addition to the covalent bond with the protein) AND it was commercially available. Visual inspection continued until a total of ten compounds were picked.

To find additional K2 analogues, K2 was split into its building blocks through its amide bond, resulting in an amine (K2-NH2) and a carboxylic acid (K2-COOH). Several vendors were searched for commercially available amines and carboxylic acids that were similar to K2-NH2 and K2-COOH (see Figures S1 and S2). Amine compounds that were picked contained a 1,3-benzodioxole motif and had a Tanimoto similarity^38^ with a value of at least 0.6 compared to K2-NH2, while picked carboxylic acid compounds contained a piperidine motif and had a Tanimoto similarity with a value of at least 0.7 compared to K2-COOH. The amines and carboxylic acids were then virtually combined via amide coupling, and the resulting compounds were prepared and covalently docked the same way as described for the Enamine Acrylamides library. The results were ranked based on DS. Building block analysis was done for the top 100 compounds based on docking score (Figure S3), and ultimately, one compound (A14C2) was synthesized based on its binding mode, building block price, and synthetic availability.

### Procedures

NMR measurements were performed on a System 300 NMR spectrometer (Varian, Palo Alto, CA, USA). ^1^H and ^13^C NMR spectra were measured at room temperature (25 °C) in an appropriate solvent. ^1^H and ^13^C chemical shifts are expressed in parts per million (δ) referenced to TMS or residual solvent signals. All chemicals and solvents were used as purchased. HPLC-MS measurements were performed using a LC-MS-2020 device (Shimadzu, Kyoto, Japan) equipped with a Reprospher 100 C18 (5 μm, 100 × 3 mm) column and positive–negative double ion source (DUIS±) with a quadrupole mass spectrometer in the range of 50–1000 *m*/*z*. Samples were eluted with gradient elution using eluent A (0.1% formic acid in water) and eluent B (0.1% formic acid in acetonitrile). Flow rate was set to 1.5 mL/min. The initial condition was 0% B eluent, followed by a linear gradient to 100% B eluent by 2 min, from 2 to 3.75 min 100% B eluent was retained, and from 3.75 to 4.5 min back to initial condition and retained to 5 min. The column temperature was kept at 30 °C, and the injection volume was 1 μL.

### Fluorescent Polarization (FP) Assay

The STAT3 protein (residues 127–688) was expressed and purified as described in our recent work.^39^ Fluorescence polarization assays were performed on a Molecular Devices SpectraMax iD5Multimode Microplate Reader (San Jose, CA, USA) using Greiner black 384-well flat-bottom nonbinding microplates with 40 μL final well volumes. The fluorescent peptide (5-FAM-G­(pTyr)­LPQTV-NH_2_, purchased from GenScript Biotech Ltd., Piscataway, NJ, USA), as well as the protein were diluted with a buffer containing 50 mM NaCl, 10 mM HEPES (4-(2-hydroxyethyl)-1-piperazineethanesulfonic acid), 1 mM EDTA (ethylenediaminetetraacetic acid), 2 mM DTT (dithiothreitol), and 0.1% Triton X-100, pH 7.5. The final concentration of the STAT3 protein was 200 nM, and the fluorescent peptide was added at a final concentration of 5 nM. The wells were treated with varying concentrations of inhibitor compounds, with a 5% final DMSO content. Protein and inhibitors were incubated at 37 °C for 1 h. Then the fluorescence peptide was added, and then the plate was incubated for another 20 min prior to the fluorescence readout (extinction wavelength: 475 nm, emission wavelength: 520 nm). The measurements were carried out using 3 parallel biological replicas. Fluorescence polarization was calculated from the perpendicular and parallel fluorescence intensities and then plotted against concentration. The IC_50_ values were determined after fitting quadratic dose–response curves on data points using GraphPad Prism 8.0.1. software.

### Safety

The GHS Category 1 corrosive chemicals trifluoracetic acid, acryloyl chloride, propionyl chloride, 2-chloroethanesulfonyl chloride, triethylamine, and *N*,*N*-diisopropylethylamine constitute significant safety hazards and must be handled with extreme care. Full synthetic protocols are reported in the Supporting Information.

## Supplementary Material



## Notes

Structures of the relevant building blocks and their occurrences in virtual screening results (Figures S1–S3); detailed synthesis and characterization (^1^H and ^13^C NMR spectra, HRMS spectra, HPLC traces) of compounds, intact protein MS, and peptide mapping protocols; detailed results of intact MS, peptide mapping, and FP assay measurements (Figures S4–S6) (DOCX)

## Abbreviations

CCD: coiled-coil domain
CNS: central nervous system
DBD: DNA-binding domain
DS: docking score
FP: fluorescence polarization
LC: liquid chromatography
MS: mass spectrometry
SH2: Src homology 2 [domain]
STAT: signal transducer and activator of transcription
TCI: targeted covalent inhibitor
VS: virtual screening
