# Supplementary material for: Allosteric Covalent Inhibitors of the STAT3 Transcription Factor from Virtual Screening
Source: ACS Med Chem Lett. 2025 May 6;16(6):991–7. doi: 10.1021/acsmedchemlett.4c00622 (PMC12169489; doi:10.1021/acsmedchemlett.4c00622)

**Supporting information**

Allosteric covalent inhibitors of the STAT3 transcription factor from virtual screening

*Tibor Viktor Szalai*^1,2,3,§^*, Vincenzo di Lorenzo* ^1,2,4,§^*, Nikolett Péczka*^1,2,4,§^*, Levente M. Mihalovits^1,2^, László Petri^1,2^, Qirat F. Ashraf^5^, Elvin D. de Araujo^6^, Viktor Honti^7^, Dávid Bajusz^1,2^, György M. Keserű*^1,2,4,*^

*For the list of affiliations, see the main text.*

Table of Contents

[Figure S1. Commercially available amine building blocks similar to K2-NH2. 2](#_Toc194960915)

[Figure S2. Commercially available carboxylic acid building blocks similar to K2-COOH. 3](#_Toc194960916)

[Figure S3. Occurrence of each amine and carboxylic acid building block within the top 100 compounds based on DS out of the 330 K2-analogues. 4](#_Toc194960917)

[Synthesis of K2 analogues 5](#_Toc194960918)

[Intact protein LC-MS analysis 8](#_Toc194960919)

[Peptide mapping 8](#_Toc194960920)

[Supplementary Characterization Data 8](#_Toc194960921)

[Figure S4. Intact MS labelling of the compounds 27](#_Toc194960922)

[Figure S5. Digestion results 29](#_Toc194960923)

[Figure S6. Fluorescence polarization assay results 31](#_Toc194960924)

# **Figure S1.** Commercially available amine building blocks similar to K2-NH2.


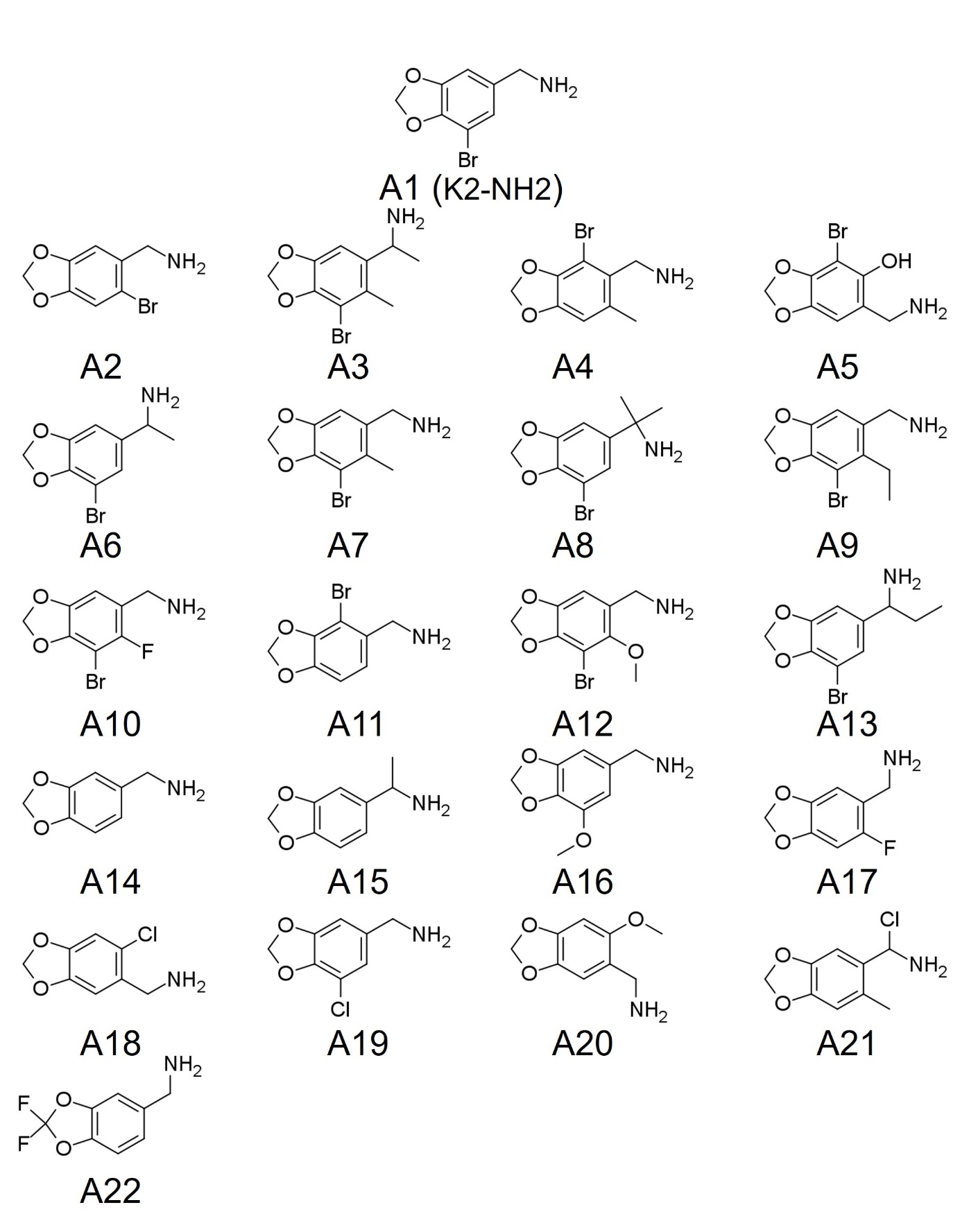


# **Figure S2.** Commercially available carboxylic acid building blocks similar to K2-COOH.


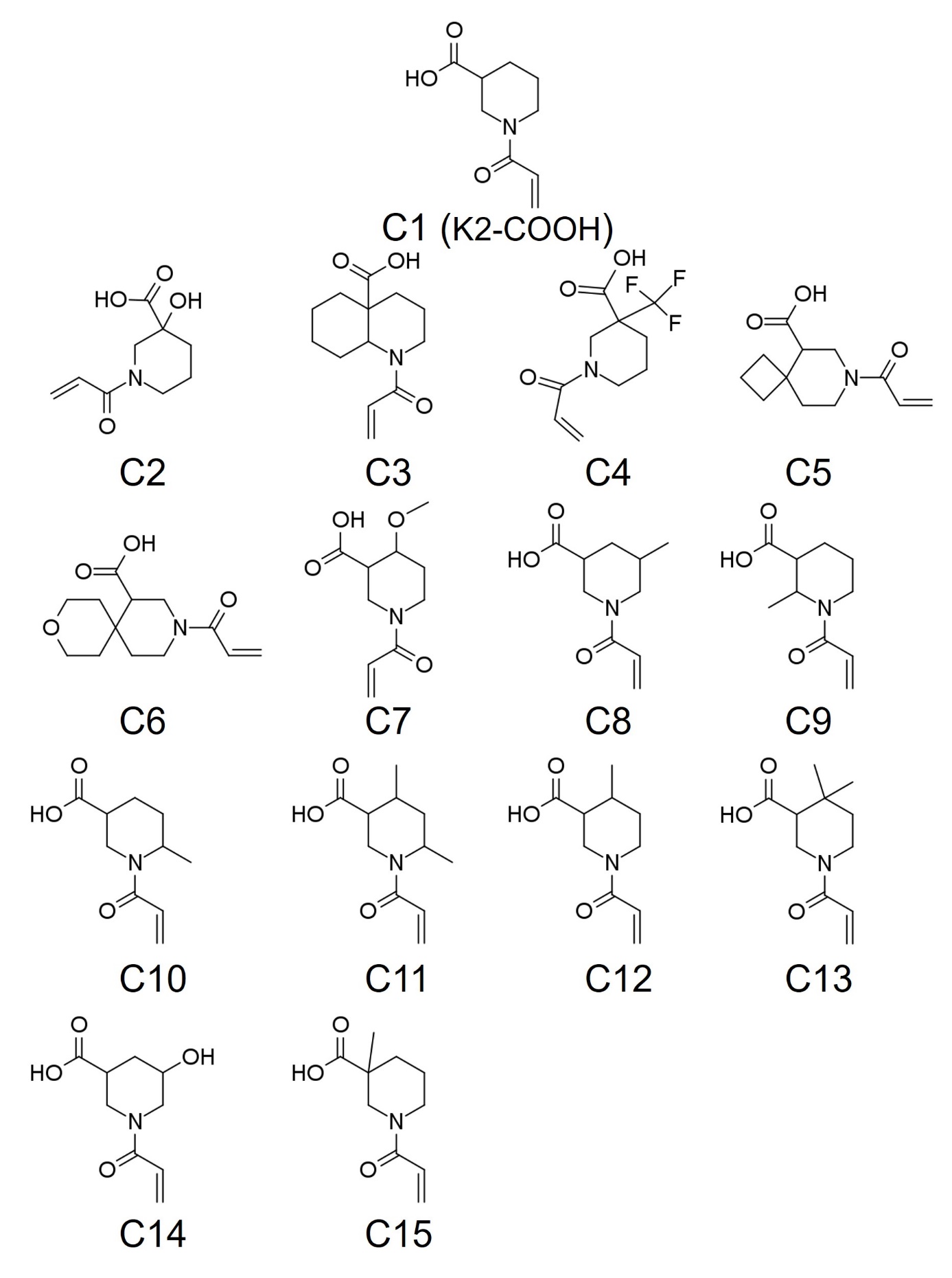


# **Figure S3.** Occurrence of each amine and carboxylic acid building block within the top 100 compounds based on DS out of the 330 K2-analogues.


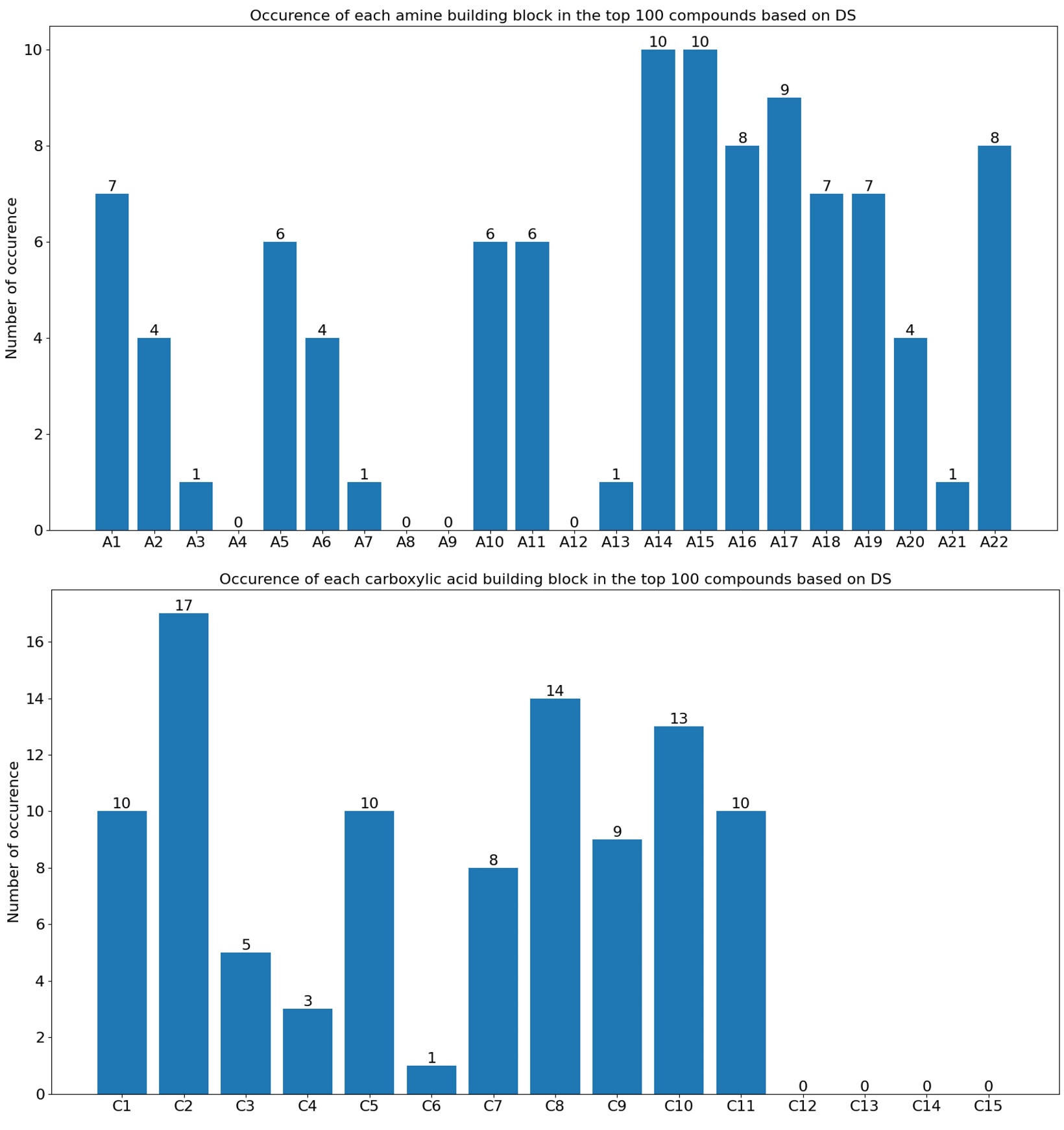


# **Synthesis of K2 analogues**

**Safety Statement.** Trifluoracetic, propionyl chloride, triethylamine and *N,N*-diisopropylethylamine cause acid causes severe skin burns and eye damage. Acryloyl chloride may be corrosive to metals, causes severe skin burns and eye damage and is fatal if inhaled. 2-Chloroethanesulfonyl chloride causes severe skin burns and eye damage and is fatal if inhaled.

*tert*-butyl 3-((benzo[d][1,3]dioxol-5-ylmethyl)carbamoyl)piperidine-1-carboxylate (**3a**)

(*S*)-1-Boc-nipecotic acid (**2a**) (1.15 g, 5 mmol) was dissolved in 10 mL MeCN, then piperonyl amine (**1**) (0.62 mL, 5 mmol), HATU (1.90 g, 5mmol) and DIPEA (1.74 mL, 10 mmol) were added. The resulting mixture was stirred at room temperature overnight. Then 15 mL water was added and extracted with dichloromethane. The combined organic layers were dried with Na_2_SO_4_, then the solvents were evaporated *in vacuo*. The crude product was purified via reverse phase flash-chromatography, resulting in 1.3 g (3.6 mmol, 72% yield) pure product.

^1^H NMR (300 MHz, CDCl_3_) δ 6.78 – 6.65 (m, 3H), 5.93 (s, 2H), 4.31 (d, *J* = 5.5 Hz, 2H), 4.01 – 3.58 (m, 2H), 3.31 – 2.84 (m, 2H), 2.29 (s, 1H), 1.97 – 1.77 (m, 2H), 1.70 – 1.55 (m, 2H), 1.41 (s, 9H) ppm.

^13^C NMR (75 MHz, CDCl_3_) δ 172.78, 154.86, 147.93, 147.87, 146.92, 132.09, 121.05, 108.36, 108.27, 101.01, 79.93, 43.33, 42.93, 28.35, 28.20, 27.65, 24.13 ppm.

HRMS (ESI): calc’d for C_19_H_27_N_2_O_5_^+^ [M^+^H]^+^ 363.1919 found 363.1913

*tert*-butyl 3-((benzo[d][1,3]dioxol-5-ylmethyl)carbamoyl)-3-hydroxypiperidine-1-carboxylate (**3b**)

1-Boc-3-hydroxypiperidine-3-carboxylic acid (**2b**) (0.05 g, 0.2 mmol) was dissolved in 2 mL MeCN, then piperonyl amine (**1**) (0.025 mL, 0.2 mmol), HATU (0.08 g, 0.22 mmol) and DIPEA (0.07 mL, 0.4 mmol) were added. The resulting mixture was stirred at room temperature overnight. Then 3 mL water was added and extracted with dichloromethane. The combined organic layers were dried with Na_2_SO_4_, then the solvents were evaporated *in vacuo*. The crude product (0.07g, 92% crude yield) was used without further purification.

*N*-(benzo[*d*][1,3]dioxol-5-ylmethyl)piperidine-3-carboxamide (**4a**)

**3a** (1.3 g, 3.6 mmol) was dissolved in 10 mL dichloromethane, then trifluoracetic acid (2 mL, 20 V/V%) was added. The resulting mixture was stirred at room temperature for 2 hours. Then cc. NaHCO_3_ was added (pH 12), and the mixture was extracted with dichloromethane. The combined organic layers were dried with Na_2_SO_4_, then the solvents were evaporated *in vacuo*, resulting in 0.9 g (3.42 mmol, 95% yield) yellow solid.

^1^H NMR (300 MHz, CDCl_3_) δ 7.78 (s, 1H), 6.83 – 6.69 (m, 3H), 5.93 (s, 2H), 4.35 (d, *J* = 5.7 Hz, 2H), 3.12 – 2.90 (m, 2H), 2.94 – 2.70 (m, 2H), 2.48 – 2.37 (m, 2H), 1.98 – 1.83 (m, 1H), 1.82 – 1.42 (m, 2H) ppm.

^13^C NMR (75 MHz, CDCl_3_) δ 174.85, 147.83, 146.75, 132.65, 120.81, 108.23, 108.20, 100.98, 48.32, 46.39, 42.97, 41.94, 27.56, 23.44 ppm.

HRMS (ESI): calc’d for C_14_H_19_N_2_O_3_^+^ [M^+^H]^+^ 263.1395 found 263.1388

*N*-(benzo[*d*][1,3]dioxol-5-ylmethyl)-3-hydroxypiperidine-3-carboxamide (**4b**)

**3b** (0.07 g, 0.19 mmol) was dissolved in 2 mL dichloromethane, then trifluoracetic acid (0.4 mL, 20 V/V%) was added. The resulting mixture was stirred at room temperature for 2 hours. Then cc. NaHCO_3_ was added (pH 12), and the mixture was extracted with dichloromethane. The combined organic layers were dried with Na_2_SO_4_, then the solvents were evaporated *in vacuo*, resulting in 0.05 g (0.06 mmol, 87% crude yield) crude product, which was used without further purification.

1-acryloyl-*N*-(benzo[*d*][1,3]dioxol-5-ylmethyl)piperidine-3-carboxamide (**6a**)

**4a** (0.33 g, 1.25 mmol) was dissolved in 5 mL dichloromethane, then TEA (0.19 mL, 1.38 mmol) and acryloyl-chloride (0.11 mL, 1.38 mmol) were added at 0°C. The resulting mixture was stirred at room temperature for 30 minutes. 7 mL water was added, and the mixture was extracted with dichloromethane. The combined organic layers were dried with Na_2_SO_4_, then the solvents were evaporated *in vacuo*. The crude product was purified via reverse phase flash-chromatography, resulting in 0.25 g (0.79 mmol, 63% yield) pure product.

^1^H NMR (300 MHz, CDCl_3_) δ 6.95 (s, 1H), 6.71 (d, *J* = 6.7 Hz, 3H), 6.51 (dd, *J* = 16.9, 10.5 Hz, 1H), 6.28 – 6.11 (m, 1H), 5.91 (s, 2H), 5.65 (dd, *J* = 10.4, 1.9 Hz, 1H), 4.40 – 4.16 (m, 2H), 4.05 (d, *J* = 13.8 Hz, 1H), 3.73 – 3.23 (m, 2H), 2.71 (t, *J* = 12.1 Hz, 1H), 2.47 – 2.18 (m, 1H), 2.17 – 1.92 (m, 1H), 1.92 – 1.56 (m, 2H), 1.56 – 1.38 (m, 1H) ppm.

^13^C NMR (75 MHz, CDCl_3_) δ 172.21, 165.73, 147.76, 146.82, 132.26, 128.23, 127.28, 121.06, 108.37, 108.21, 100.97, 46.51, 44.20, 43.30, 42.49, 27.44, 24.88 ppm.

HRMS (ESI): calc’d for C_17_H_21_N_2_O_4_^+^ [M^+^H]^+^ 317.1501 found 317.1490

1-acryloyl-*N*-(benzo[*d*][1,3]dioxol-5-ylmethyl)-3-hydroxypiperidine-3-carboxamide (**6b**)

**4b** (0.046 g, 0.17 mmol) was dissolved in 2 mL dichloromethane, then acryloyl-chloride (0.014 mL, 0.17 mmol) were added. The resulting mixture was stirred for 30 minutes. Next the solvents were evaporated *in vacuo*. The crude product was purified via reverse phase flash-chromatography, resulting in 20 mg (0.06 mmol, 30% yield) pure product.

^1^H NMR (300 MHz, CDCl_3_) δ 7.44 (s, 1H), 6.75 (d, *J* = 7.3 Hz, 3H), 6.66 – 6.39 (m, 1H), 6.34 – 6.19 (m, 1H), 5.94 (s, 2H), 5.72 (d, *J* = 10.6 Hz, 1H), 4.50 (d, *J* = 14.0 Hz, 1H), 4.34 (d, *J* = 5.9 Hz, 2H), 3.97 (d, *J* = 13.6 Hz, 1H), 3.28 (d, *J* = 14.0 Hz, 1H), 3.17 (t, *J* = 12.5 Hz, 1H), 2.29 – 2.03 (m, 1H), 1.82 – 1.57 (m, 3H) ppm. Rotamers are present, and the major isomer’s signals are indicated.

^13^C NMR (75 MHz, CDCl_3_) δ 174.59, 169.10, 148.47, 147.53, 132.49, 129.47, 127.78, 121.52, 108.85, 101.61, 75.58, 50.12, 47.22, 43.57, 33.40, 22.41 ppm.

HRMS (ESI): calc’d for C_17_H_21_N_2_O_5_^+^ [M^+^H]^+^ 333.1450 found 333.1442

*N*-(benzo[*d*][1,3]dioxol-5-ylmethyl)-1-(vinylsulfonyl)piperidine-3-carboxamide (**8**)

**4a** (0.33 g, 1.25 mmol) was dissolved in 5 mL dichloromethane, then TEA (0.19 mL, 1.38 mmol) and 2-chloroethanesulfonyl chloride (0.14 mL, 1.38 mmol) were added at 0°C. The resulting mixture was stirred at room temperature for 1 hour, then TEA (0.19 mL, 1.38 mmol) was added again, the reaction was stirred at room temperature overnight. 7 mL water was added, then extracted with dichloromethane. The combined organic layers were dried with Na_2_SO_4_, then the solvents were evaporated *in vacuo*. The crude product was purified via reverse phase flash-chromatography, resulting in 0.14 g (0.4 mmol, 32% yield) pure product.

^1^H NMR (300 MHz, CDCl_3_) δ 6.80 – 6.66 (m, 3H), 6.49 – 6.33 (m, 1H), 6.22 (d, *J* = 16.6 Hz, 1H), 6.03 (d, *J* = 9.8 Hz, 1H), 5.94 (s, 2H), 4.44 – 4.22 (m, 2H), 3.73 – 3.61 (m, 1H), 3.61 – 3.50 (m, 1H), 2.99 – 2.85 (m, 1H), 2.79 – 2.64 (m, 1H), 2.52 – 2.36 (m, 1H), 1.96 – 1.84 (m, 1H), 1.87 – 1.54 (m, 3H) ppm.

^13^C NMR (300 MHz, CDCl_3_) δ 172.35, 147.88, 146.96, 132.23, 131.85, 128.84, 121.05, 108.33, 108.30, 101.05, 77.46, 77.04, 76.61, 47.87, 45.89, 43.31, 42.91, 27.28, 24.07 ppm.

HRMS (ESI): calc’d for C_16_H_21_N_2_O_5_S^+^ [M^+^H]^+^ 353.1171 found 353.1163

*N*-(benzo[*d*][1,3]dioxol-5-ylmethyl)-1-propionylpiperidine-3-carboxamide (**10a**)

**4a** (0.02 g, 0.07 mmol) was dissolved in 2 mL DMF, then HATU (0.03 g, 0.084 mmol), DIPEA (0.04 mL, 0.21 mmol) and propionic acid (0.006 mL, 0.077 mmol) were added. The resulting mixture was stirred at room temperature overnight. After the completion of the reaction, the mixture was purified via reverse phase flash-chromatography, resulting in 5.6 mg (0.02 mmol, 25% yield) pure product.

^1^H NMR (300 MHz, CDCl_3_) δ 7.43 – 7.11 (m, 1H), 6.74 – 6.51 (m, 3H), 5.80 (s, 2H), 4.43 – 3.87 (m, 3H), 3.49 (d, *J* = 13.4 Hz, 1H), 3.10 (q, *J* = 13.5, 12.4 Hz, 2H), 2.34 – 2.12 (m, 3H), 1.93 – 1.75 (m, 2H), 1.63 – 1.47 (m, 1H), 1.44 – 1.20 (m, 1H), 0.95 (t, *J* = 7.5 Hz, 3H) ppm. Rotamers are present, and the major isomer’s signals are indicated.

^13^C NMR (75 MHz, CDCl_3_) δ 173.13, 173.00, 172.95, 172.73, 147.71, 147.63, 146.73, 146.64, 132.23, 132.17, 120.85, 108.16, 108.10, 108.05, 100.95, 100.90, 47.79, 46.10, 43.99, 43.42, 43.05, 42.37, 41.94, 28.18, 27.31, 26.37, 24.70, 24.14, 21.13, 20.81, 9.39, 9.34 ppm. Rotamers are present, and all signals are indicated.

HRMS (ESI): calc’d for C_17_H_23_N_2_O_4_^+^ [M^+^H]^+^ 319.1652 found 353.1655

*N*-(benzo[*d*][1,3]dioxol-5-ylmethyl)-3-hydroxy-1-propionylpiperidine-3-carboxamide (**10b**)

**4b** (0.046 g, 0.17 mmol) was dissolved in 2 mL THF, then propionic anhydride (0.0214 mL, 0.17 mmol) was slowly added at room temerature. The resulting mixture was stirred for 30 minutes, then the solvents were evaporated *in vacuo*. The crude product was purified via reverse phase flash-chromatography, resulting in 20 mg (0.06 mmol, 35% yield) pure product.

^1^H NMR (500 MHz, CDCl_3_) δ 7.51 – 7.28 (m, 1H), 6.81 – 6.70 (m, 3H), 5.95 (s, 2H), 4.51 (d, *J* = 14.0 Hz, 1H), 4.40 – 4.31 (m, 2H), 3.87 (d, *J* = 13.8 Hz, 1H), 3.75 – 3.55 (m, 1H), 3.48 (s, 1H), 3.23 (d, *J* = 13.9 Hz, 1H), 3.14 (t, *J* = 12.3 Hz, 1H), 2.45 – 2.37 (m, 2H), 1.83 – 1.72 (m, 1H), 1.69 – 1.63 (m, 1H), 1.16 – 1.06 (m, 4H) ppm.

^13^C NMR (75 MHz, CDCl_3_) δ 175.07, 174.19, 147.87, 131.98, 120.89, 108.25, 101.01, 74.62, 50.69, 49.03, 46.13, 42.97, 32.79, 26.46, 21.47, 9.47 ppm.

HRMS (ESI): calc’d for C_17_H_23_N_2_O_5_^+^ [M^+^H]^+^ 335.1601 found 335.1610

# Intact protein LC-MS analysis

RPLC-MS analysis of the intact proteins were performed on a a Triple TOF 5600+ hybrid Quadrupole-TOF LC/MS/MS system (Sciex, MA, USA) equipped with a DuoSpray IonSource coupled with a Shimadzu Prominence LC20 UFLC (Shimadzu, Japan) system consisting of quaternary pump, an autosampler and a thermostatted column compartment. Chromatographic separation was achieved on the Discovery® BIO Wide Pore C-18-5 (250 mm × 2.1mm, 5 μm, 300 Å) HPLC column. Sample was eluted in gradient elution mode using solvent A (0.1% formic acid in water) and solvent B (0.1% formic acid in ACN). The initial condition was 5% B for 7 min, followed by a linear gradient to 90% B by 48 min, from 55 to 63 min 90% B was retained; and from 63 to 65 min back to initial condition with 5 % eluent B and retained for 10 min. Flow rate was set to 0.2 ml/min. The column temperature was 40 °C and the injection volume was 15 µl. Nitrogen was used as the nebulizer gas (GS1), heater gas (GS2), and curtain gas with the optimum values set at 35, 35 and 35 (arbitrary units), respectively. The source temperature was 350 °C and the spray voltage was set to 5000 V.

# Peptide mapping

Modification sites were determined using a Triple TOF 5600+ hybrid Quadrupole-TOF LC/MS/MS system (Sciex, MA, USA) equipped with a DuoSpray IonSource coupled with a Shimadzu Prominence LC20 UFLC (Shimadzu, Japan) system consisting of binary pump, an autosampler and a thermostatted column compartment. Data acquisition and processing were performed using Analyst TF software version 1.7.1 (Sciex Instruments, CA, USA). Chromatographic separation was achieved on a Merck BIOshellTM 400 Å Protein C18 (75 mm × 2,1mm, 3,4 µm, 400 Å) HPLC column. Sample was eluted in gradient elution mode using solvent A (0.1% formic acid in water) and solvent B (0.1% formic acid in ACN). The initial condition was 10% B for 2 min, followed by a linear gradient to 90% B by 8 min, from 10 to 12 min 90% B was retained; and from 12 to 12,5 min back to initial condition with 10 % eluent B and retained from 12,5 to 15 min. Flow rate was set to 0.5 ml/min. The column temperature was 50 °C and the injection volume was 7 µl. Nitrogen was used as the nebulizer gas (GS1), heater gas (GS2), and curtain gas with the optimum values set at 40, 45 and 40 (arbitrary units), respectively. Data were acquired in positive electrospray mode in the mass range of m/z=250 to 3000, with 1 s accumulation time. The source temperature was 400 °C and the spray voltage was set to 5000 V. Declustering potential value was set to 80 V. Peak View Software® V.2.2 (Sciex, Redwood City, CA, USA) was used for deconvoluting the raw electrospray data to obtain the neutral molecular masses.

# **Supplementary Characterization Data**

**1H and 13C NMR spectra**

**3a**


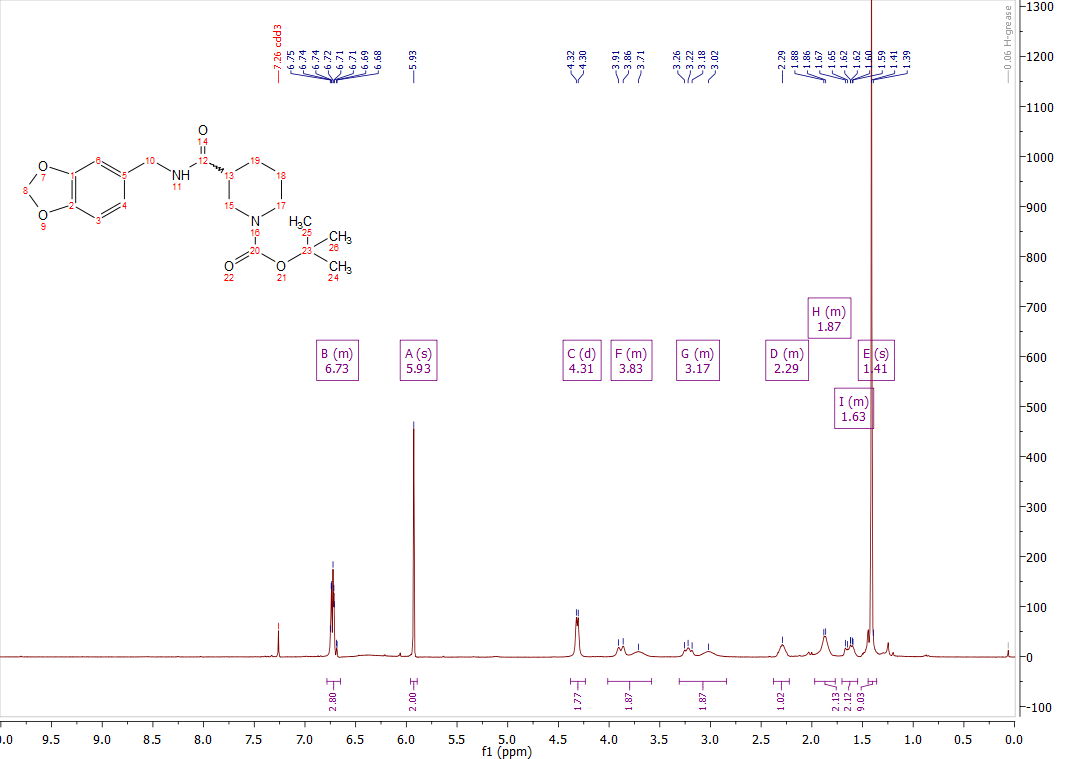


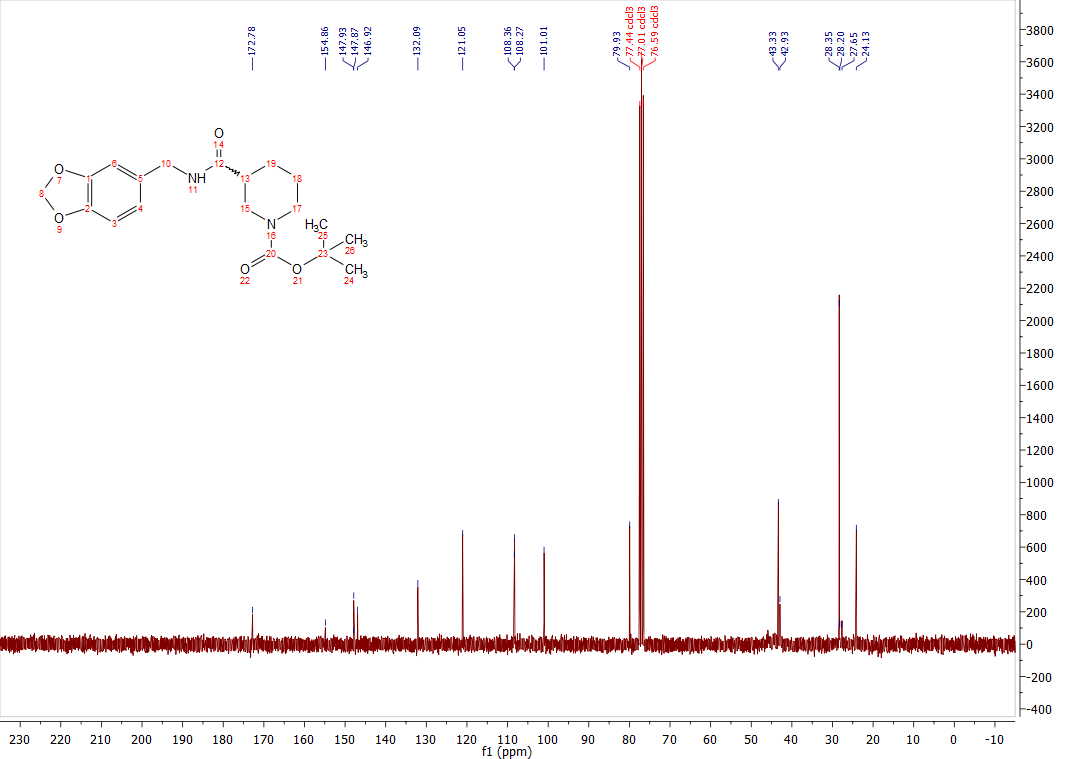


**4a**


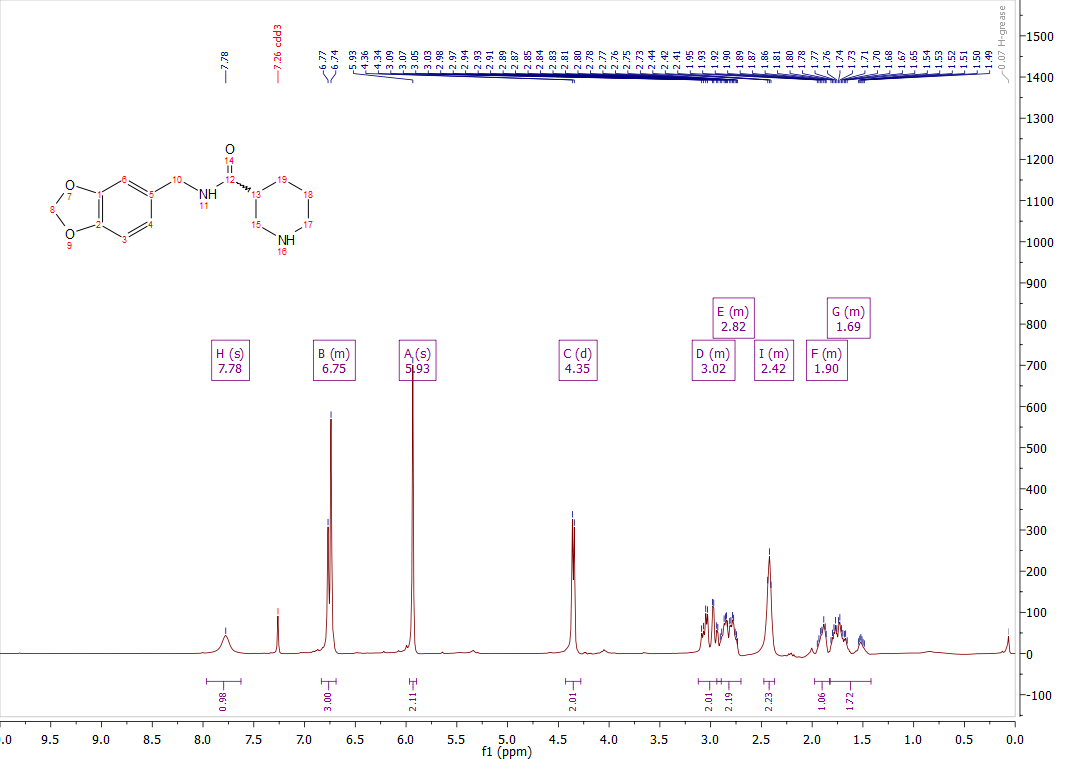

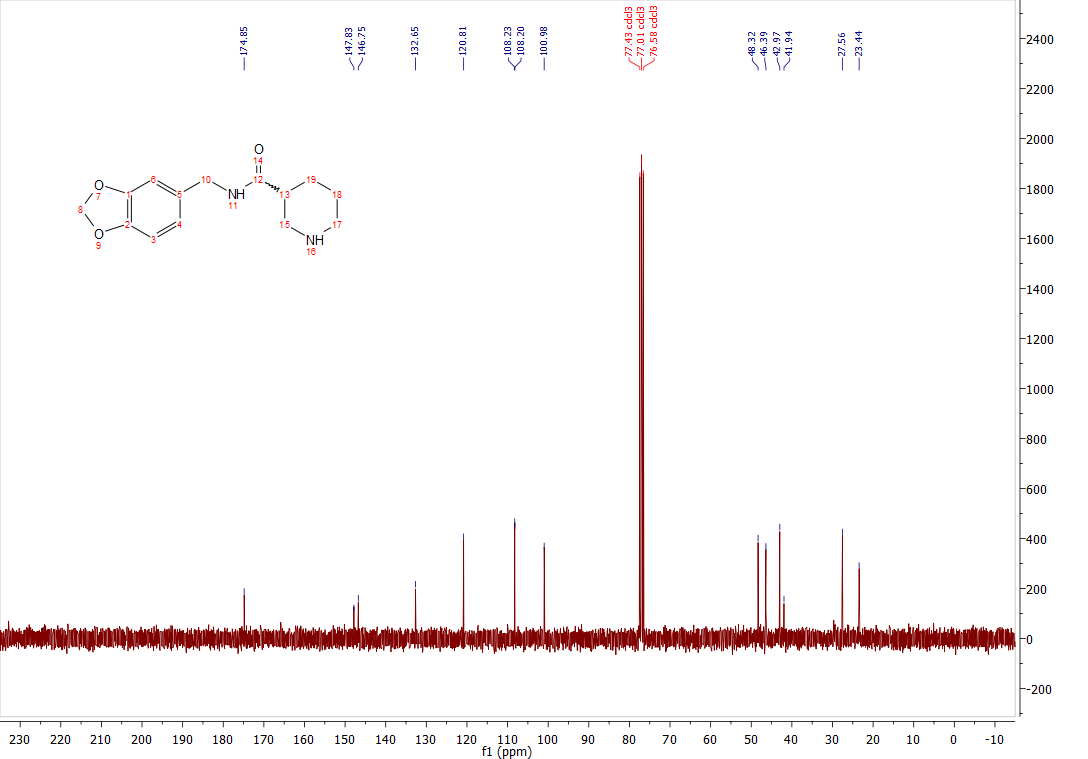


**6a**


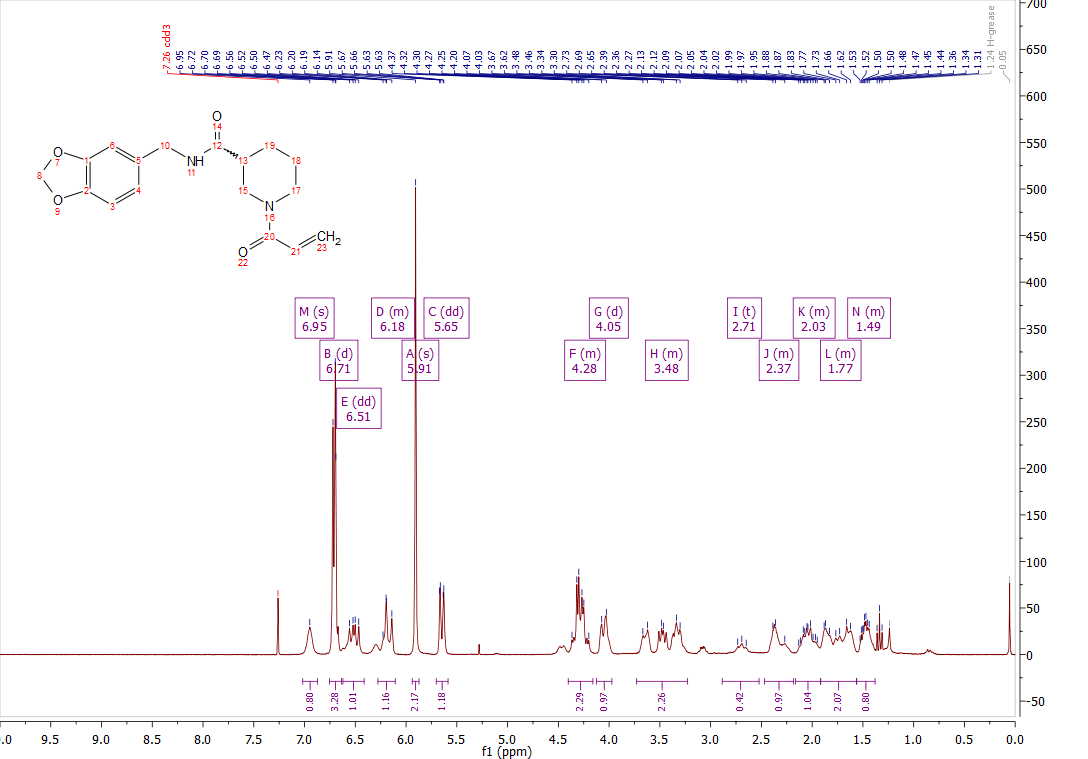

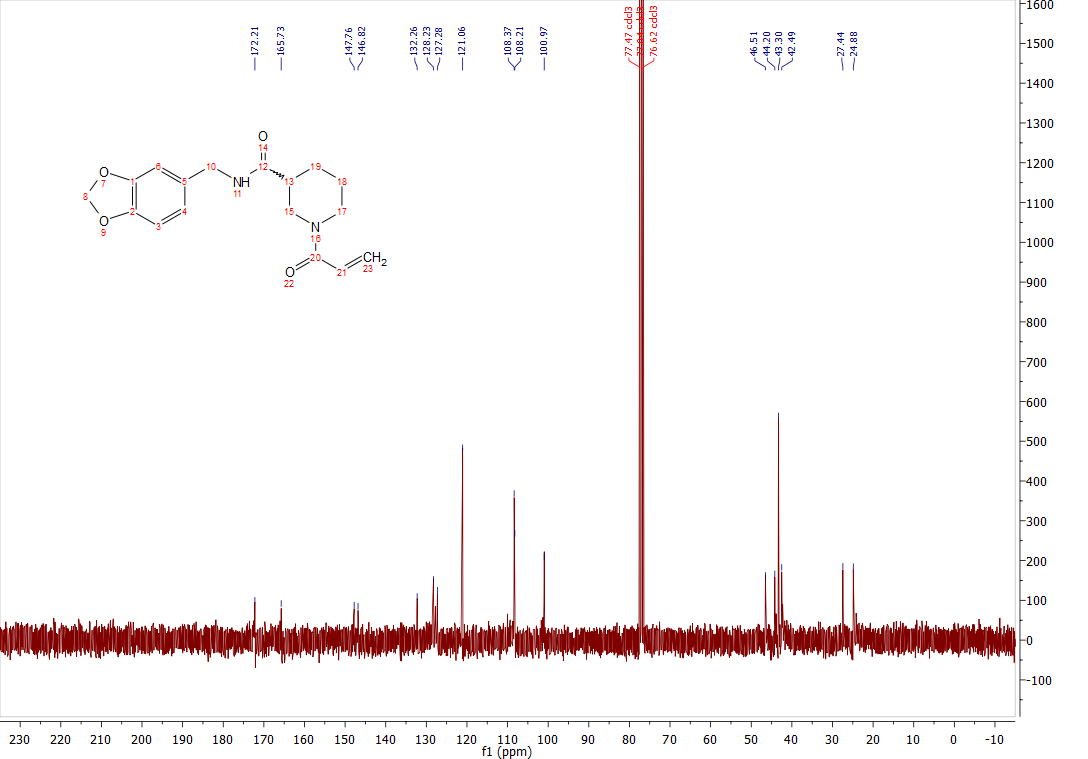


**6b**


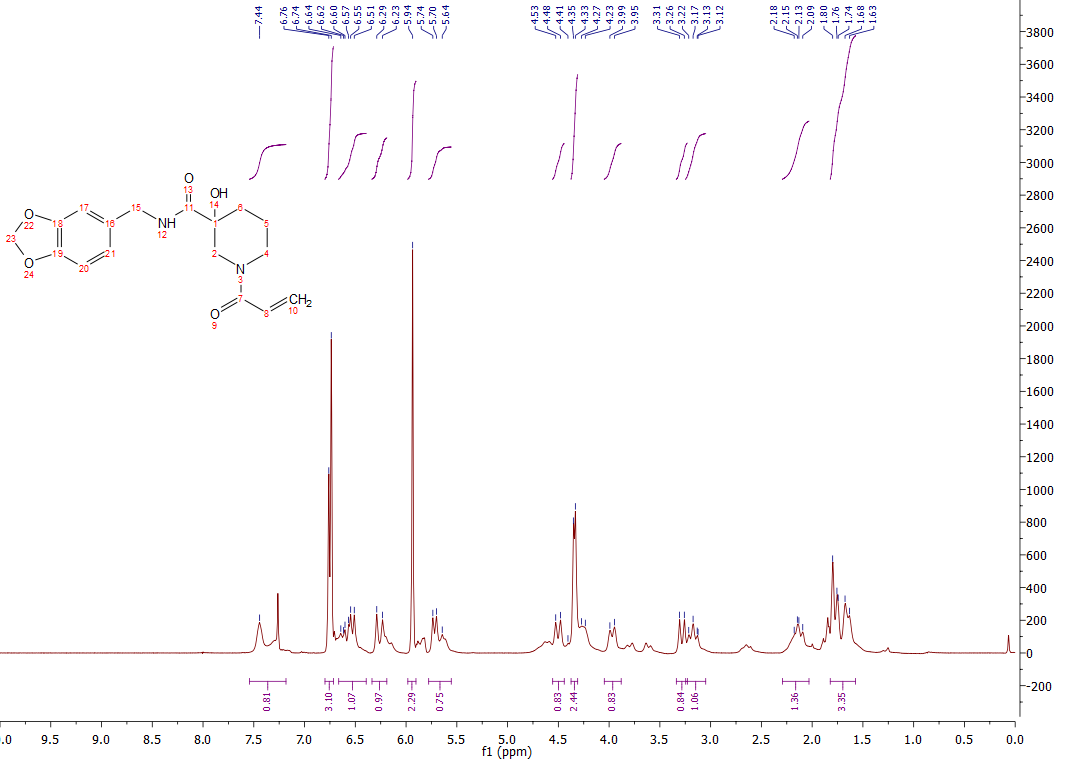

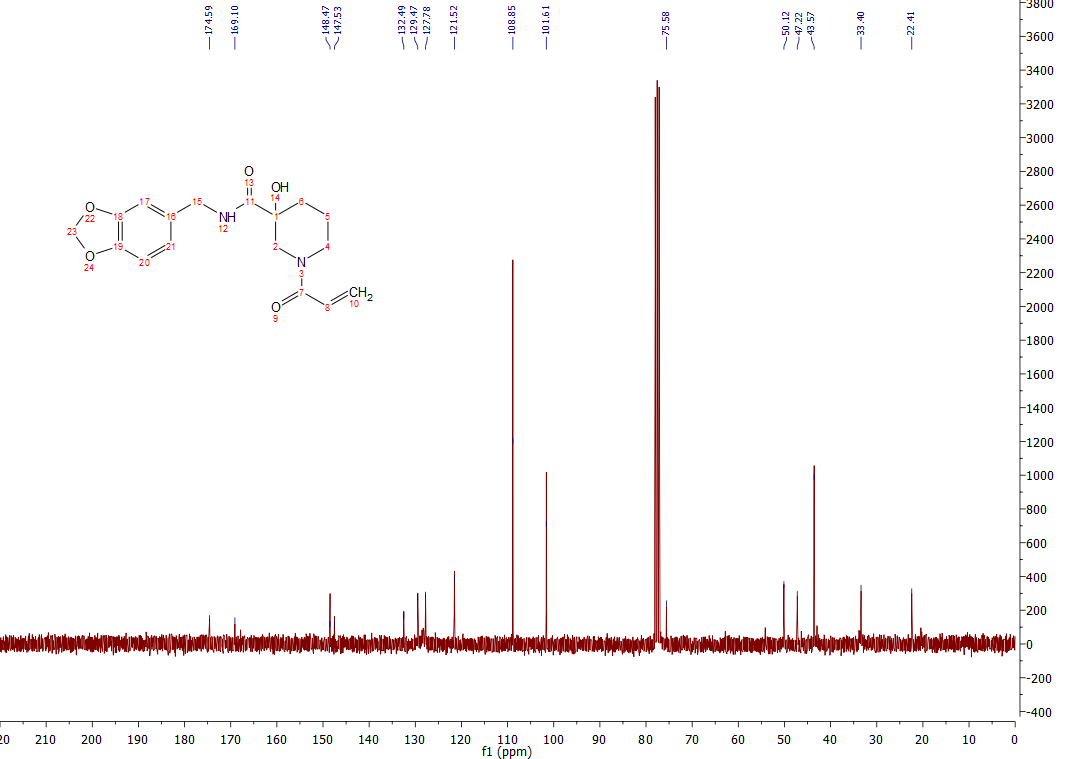


**8**


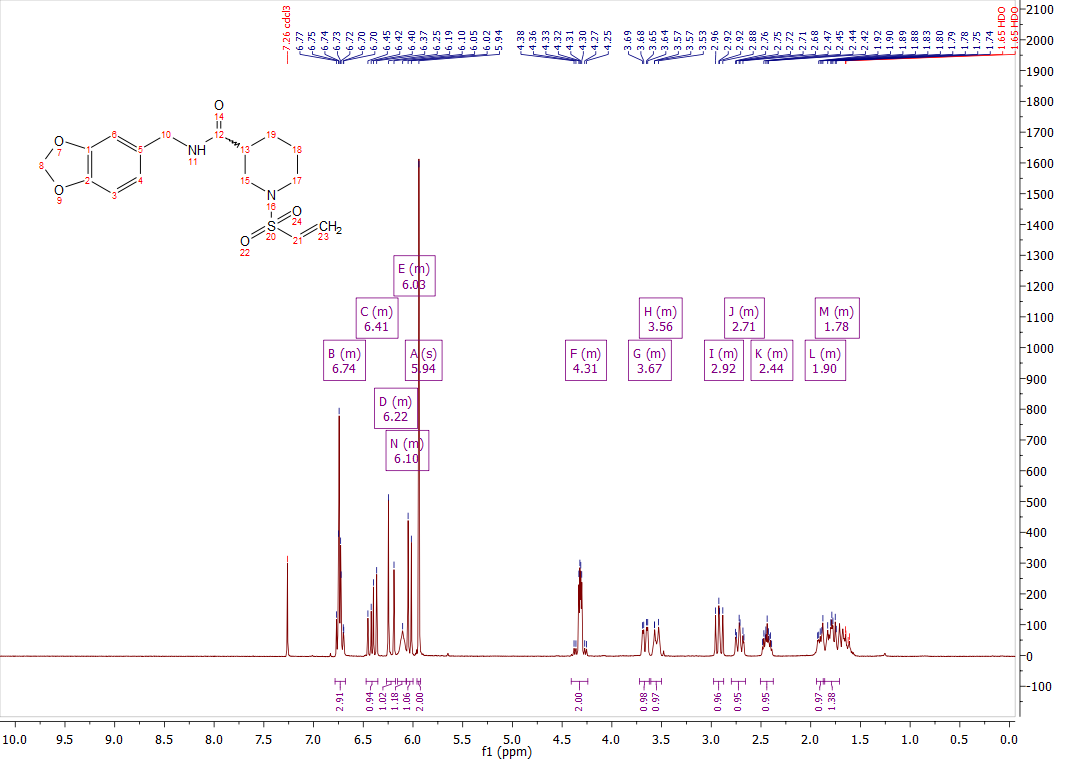

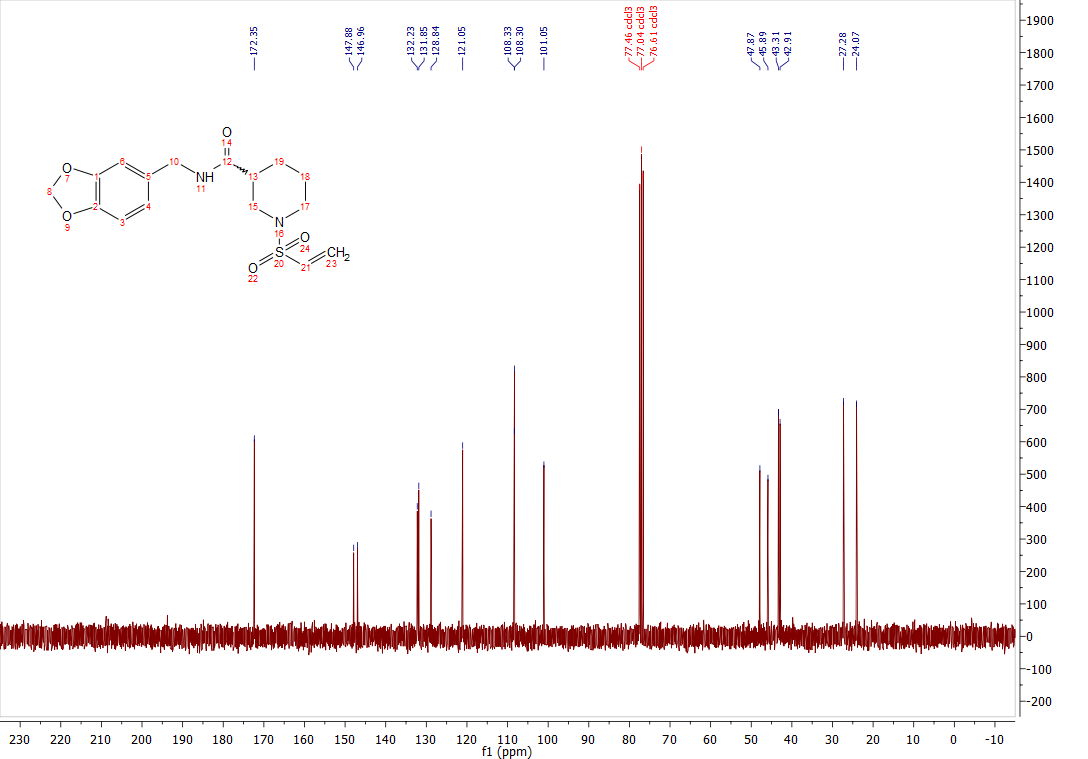


**10a**


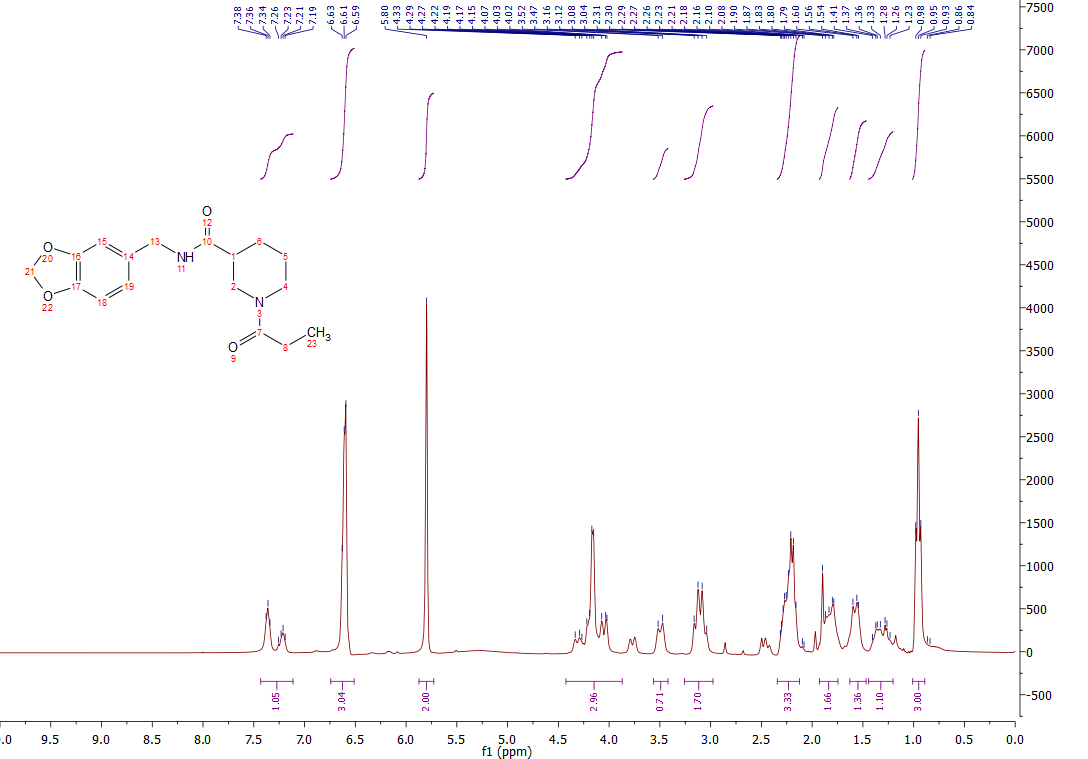

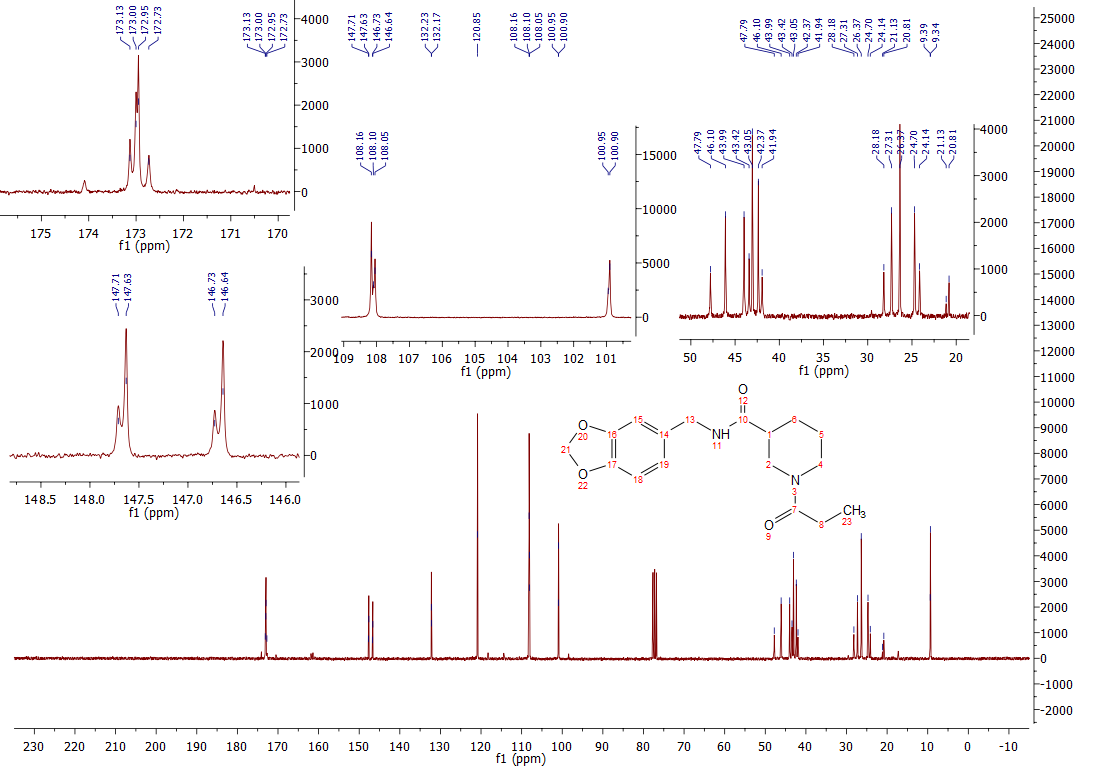


**10b**


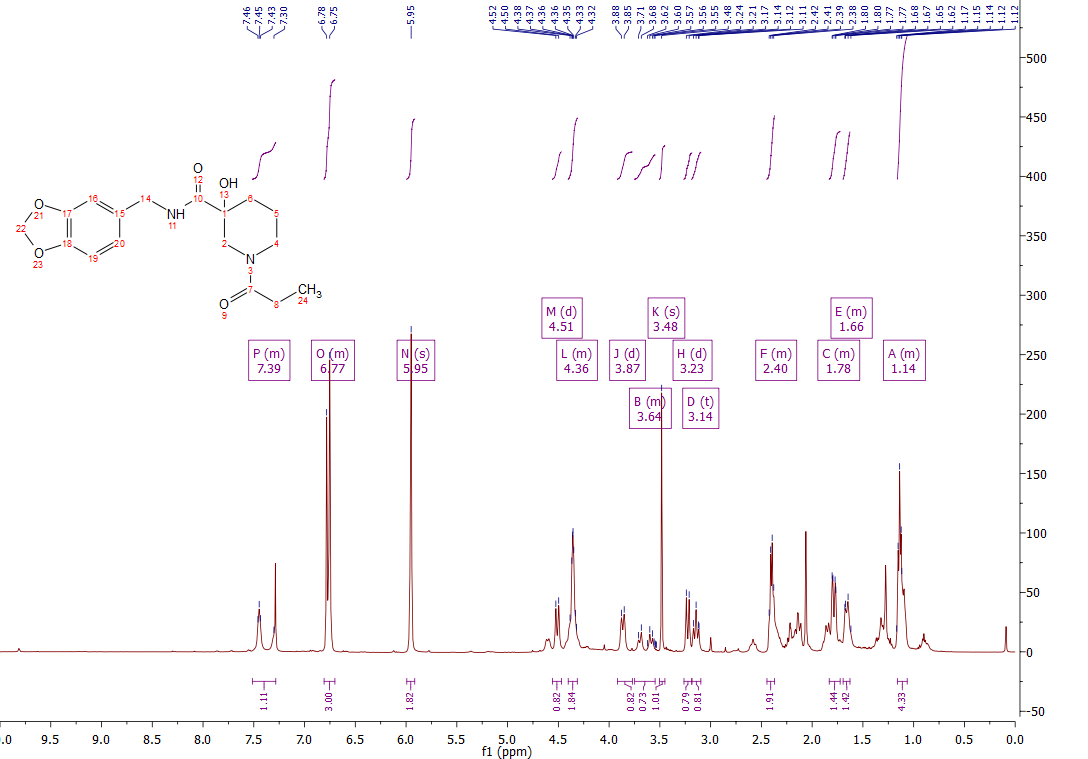

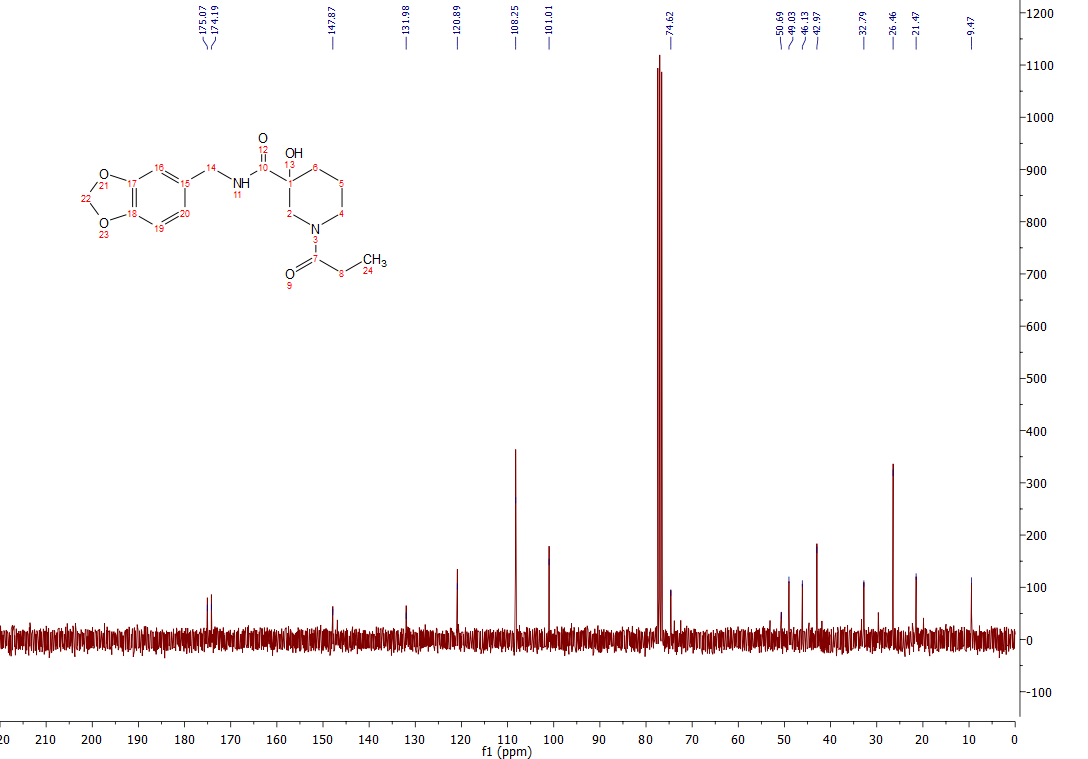


**HRMS spectra**

**3a**


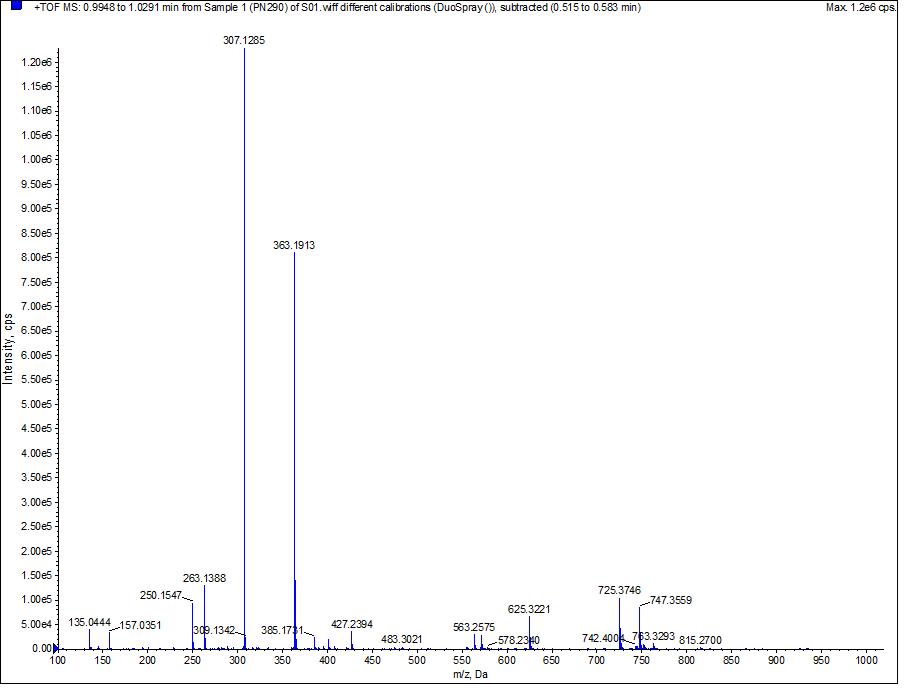


| **Formula** | **Calculated mass** | **Error / mDa** | **Error / ppm** | **DBE** |
| --- | --- | --- | --- | --- |
| C19 H27 N2 O5 | 363.1919 | -0.6972 | -1.9199 | 7.5 |
| *C15 H19 N2 O5 | 307.1293 | -0.8969 | -2.9205 | 7.5 |
| *-tBut |  |  |  |  |

**4a**


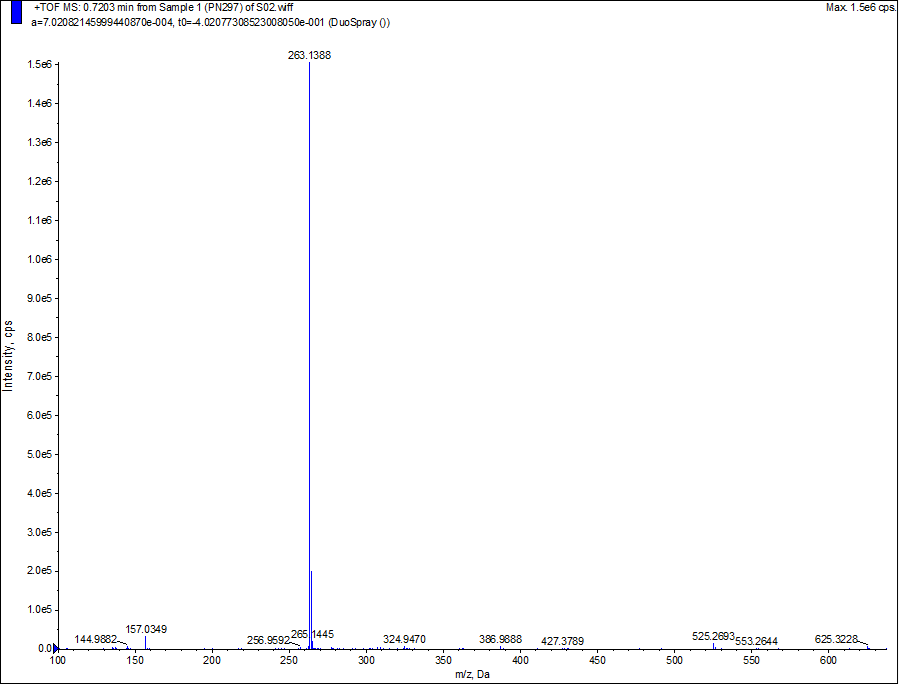


| **Formula** | **Calculated mass** | **Error / mDa** | **Error / ppm** | **DBE** |
| --- | --- | --- | --- | --- |
| C14 H19 N2 O3 | 263.1395 | -0.7676 | -2.9174 | 6.5 |

**6a**

| 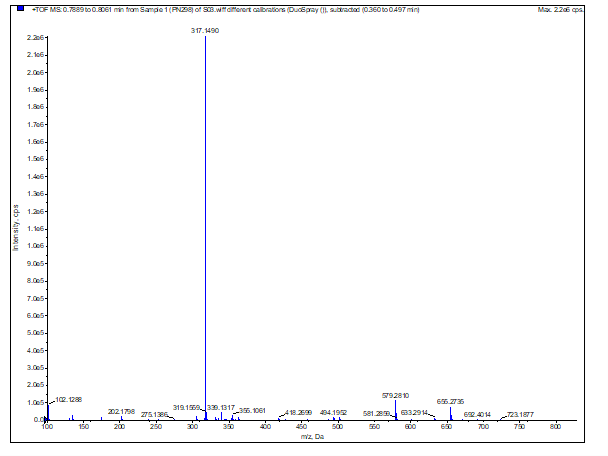 |
| --- |

| **Formula** | **Calculated mass** | **Error / mDa** | **Error / ppm** | **DBE** |
| --- | --- | --- | --- | --- |
| C17 H21 N2 O4 | 317.1501 | -1.1324 | -3.5706 | 8.5 |

**6b**


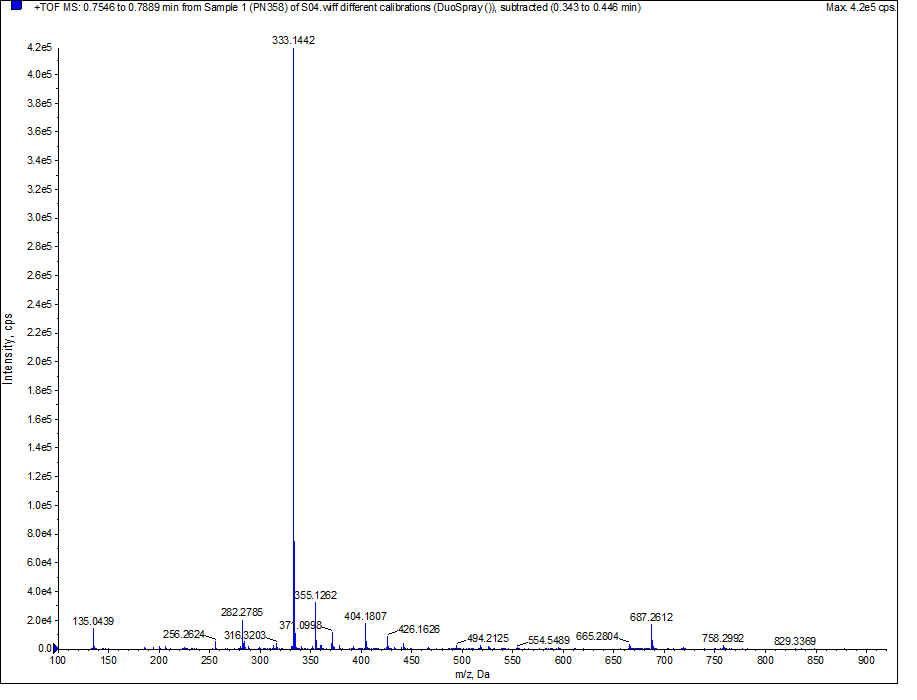


| **Formula** | **Calculated mass** | **Error / mDa** | **Error / ppm** | **DBE** |
| --- | --- | --- | --- | --- |
| C17 H21 N2 O5 | 333.145 | -0.847 | -2.5426 | 8.5 |

**8**


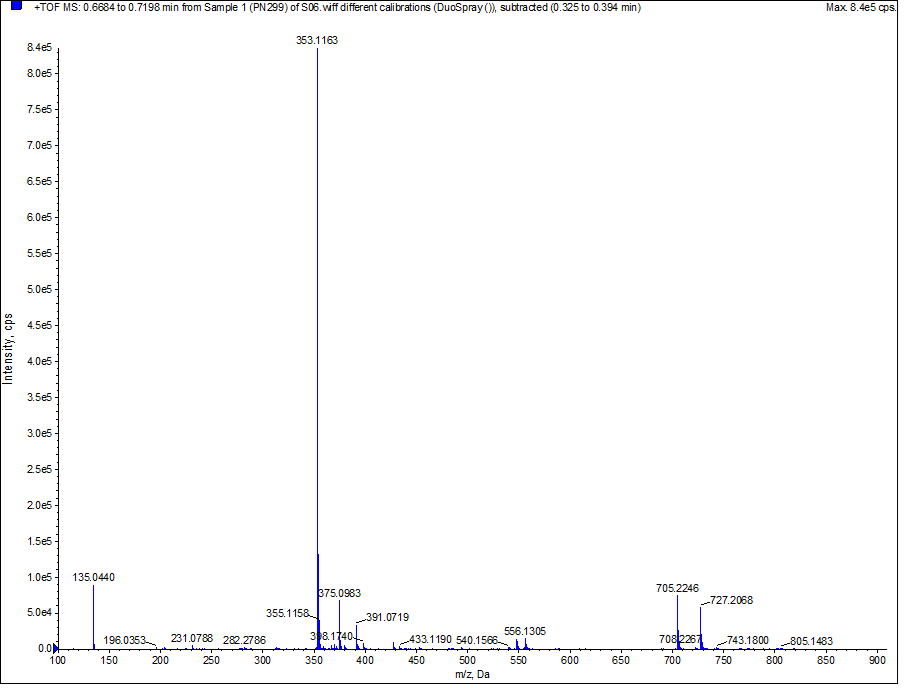


| **Formula** | **Calculated mass** | **Error / mDa** | **Error / ppm** | **DBE** |
| --- | --- | --- | --- | --- |
| C16 H21 N2 O5 S | 353.1171 | -0.8188 | -2.3189 | 7.5 |

**10a**


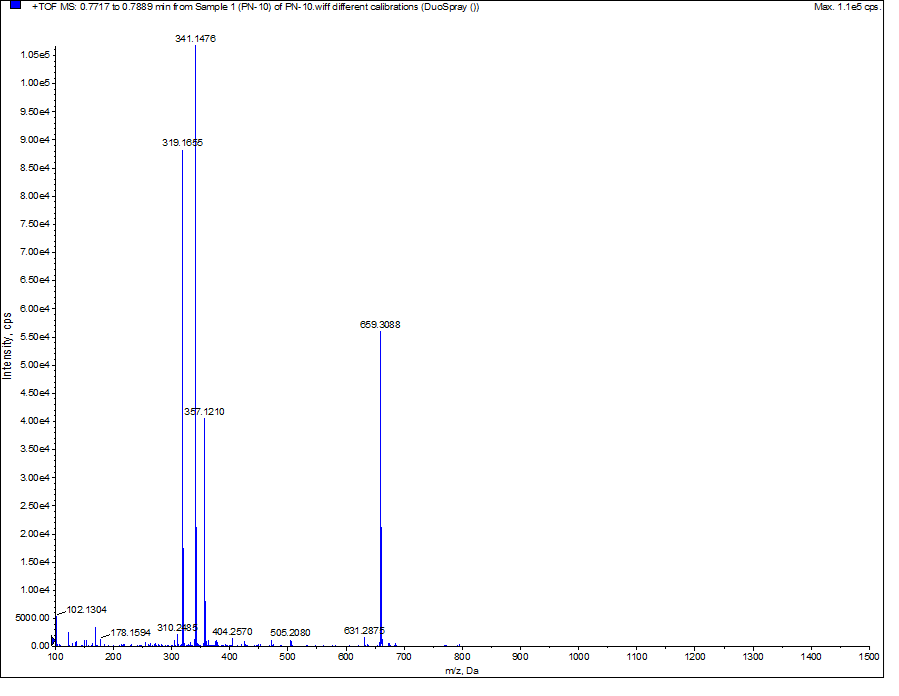


| **Formula** | **Calculated mass** | **Error / mDa** | **Error / ppm** | **DBE** |
| --- | --- | --- | --- | --- |
| C17 H23 N2 O4 | 319.1652 | 0.266 | 0.8336 | 7.5 |

**10b**


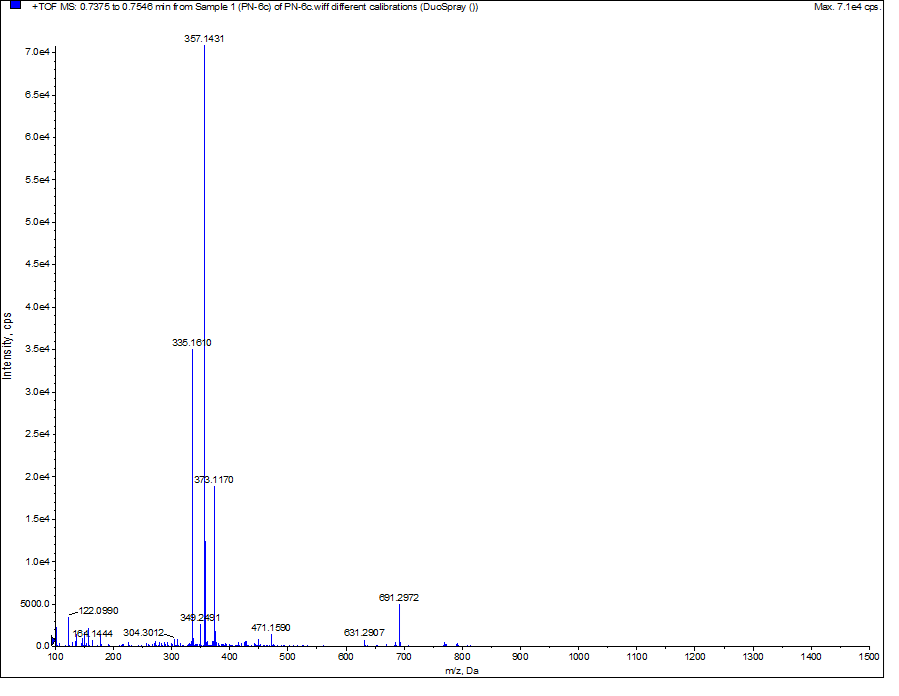


| **Formula** | **Calculated mass** | **Error / mDa** | **Error / ppm** | **DBE** |
| --- | --- | --- | --- | --- |
| C17 H23 N2 O5 | 335.1601 | 0.8514 | 2.5403 | 7.5 |

**HPLC traces of final compounds**

**6a**


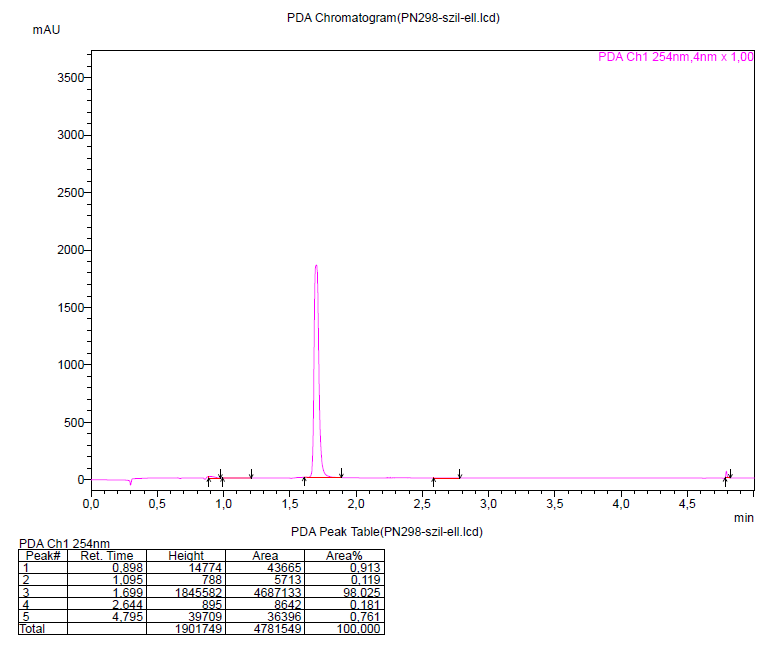


**6b**


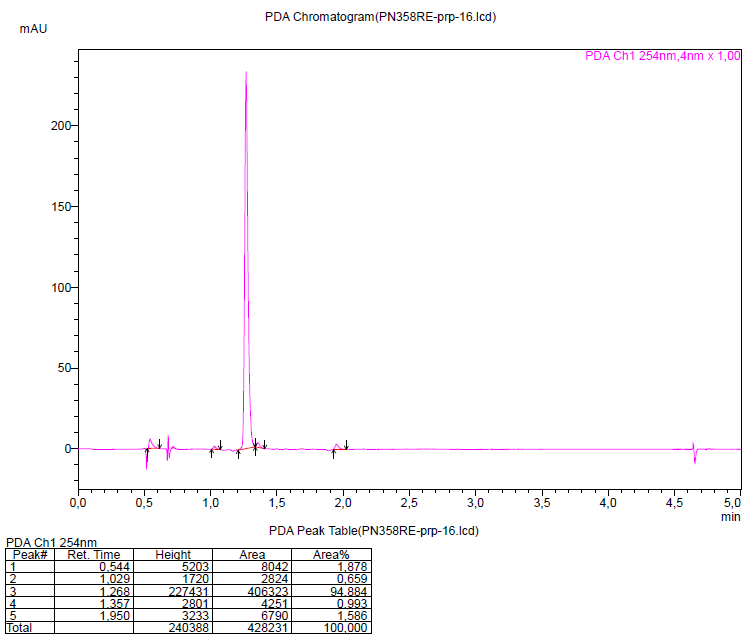


**8**


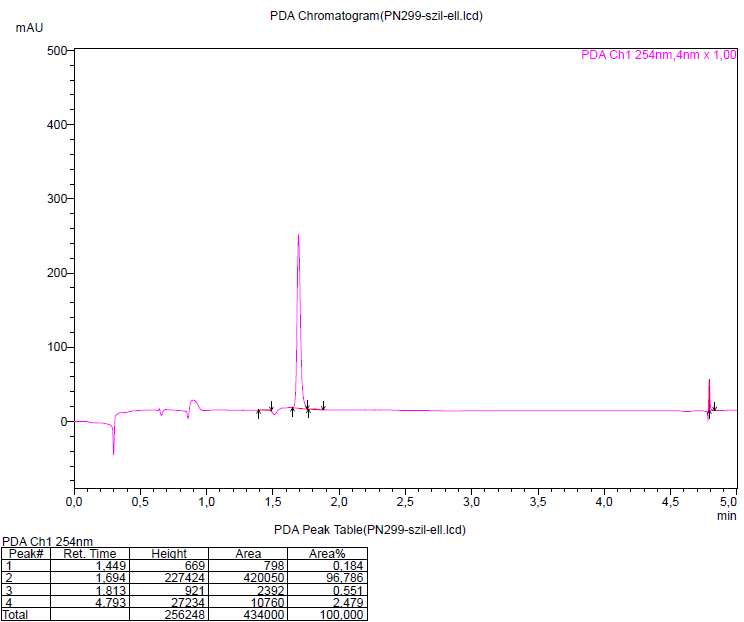


**10a**


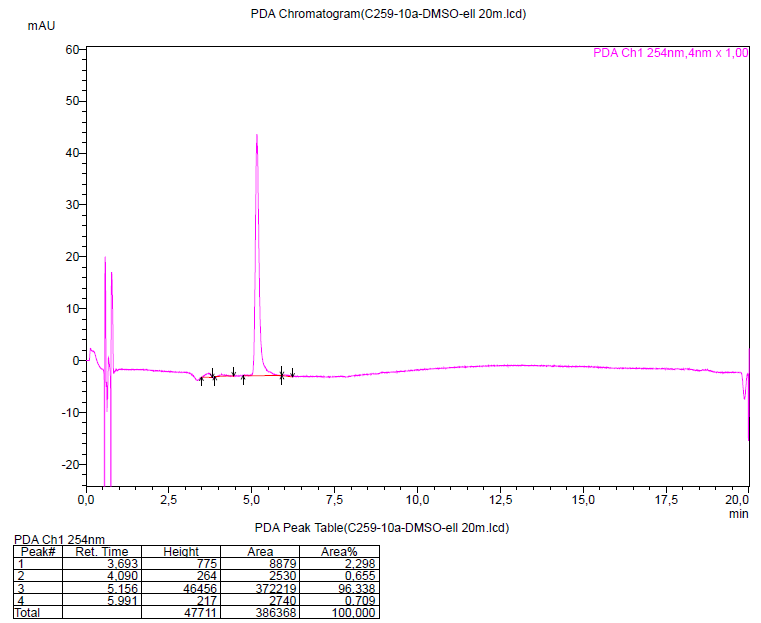


**10b**


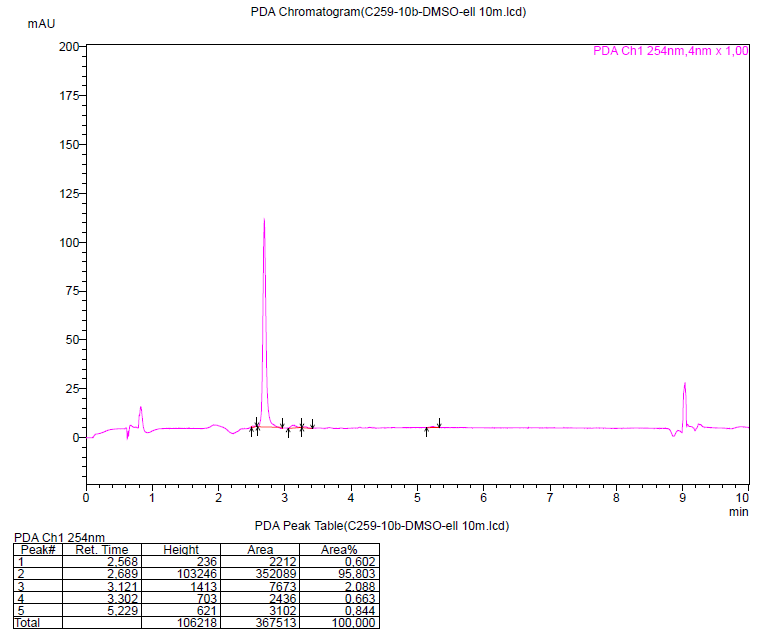


# **Figure S4**. Intact MS labelling of the compounds

**K2**


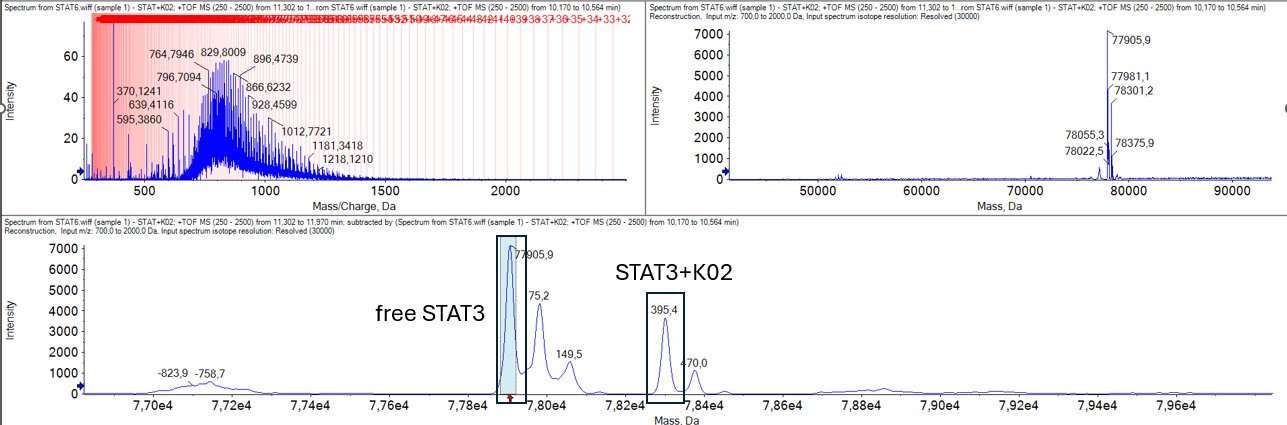


**6a**


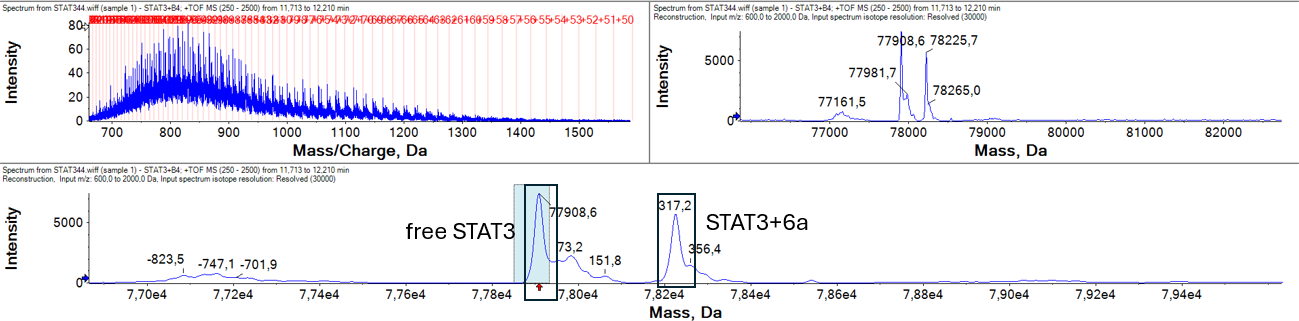


**6b**


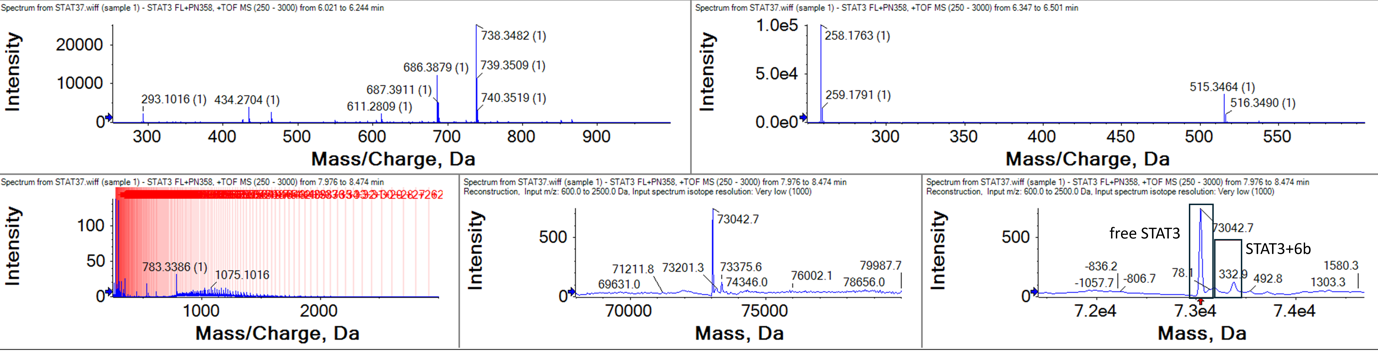


**8**


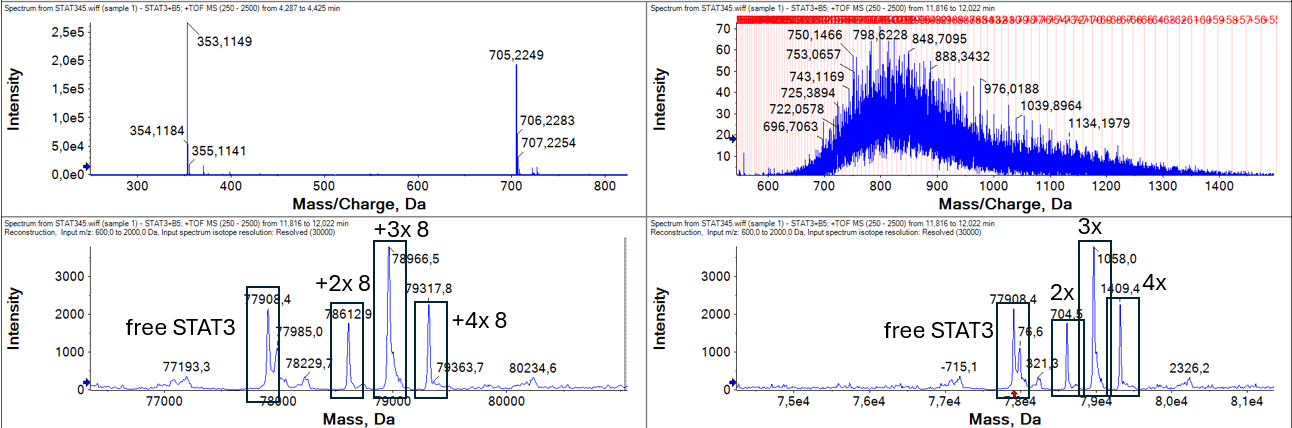


# **Figure S5**. Digestion results

**6a:**

Fragmentation of protein sequence QQIAC251[CAM]IGGPPNI**C259[6a]**LDR, and the table of theoretical (black) and found (red) fragment masses.


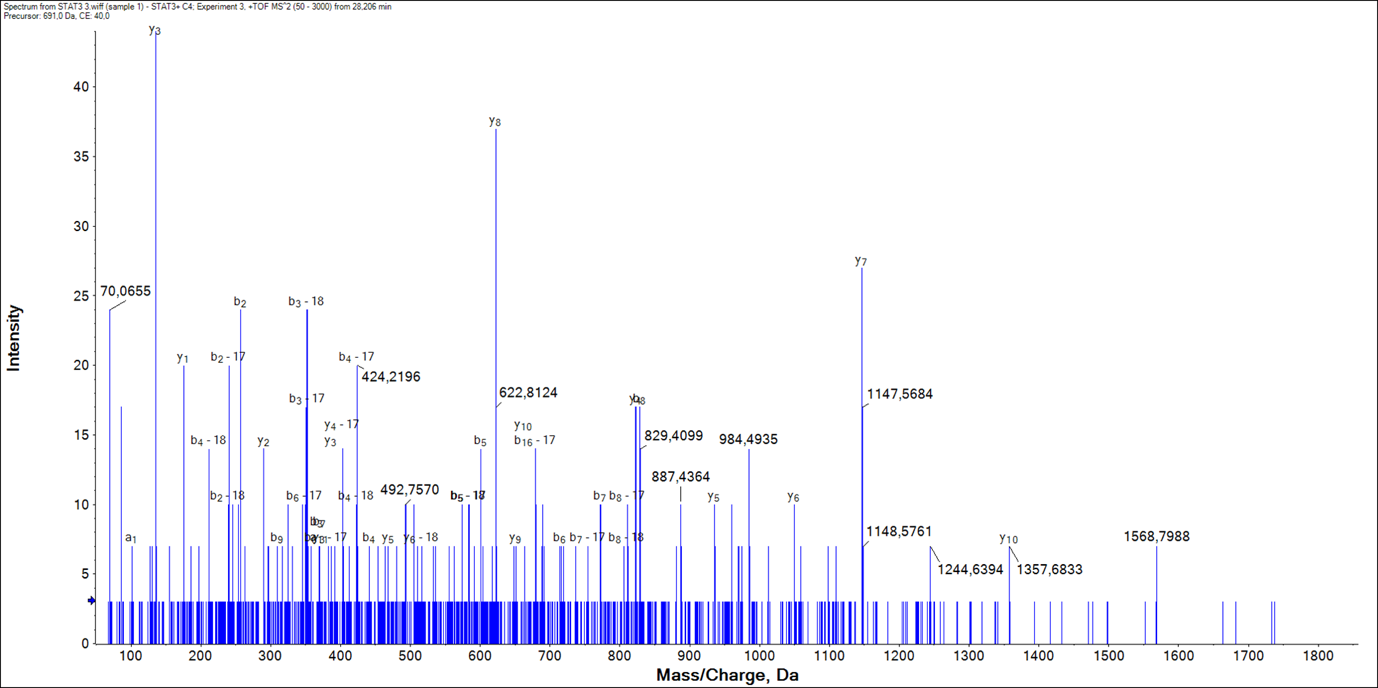

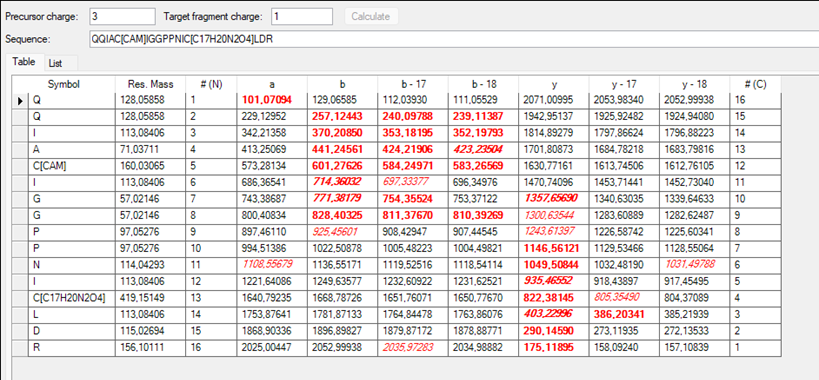


**6b:**

Fragmentation of protein sequence QQIAC251[**6b**]IGGPPNI**C259[**CAM**]**LDR, and the table of theoretical (black) and found (red) fragment masses.


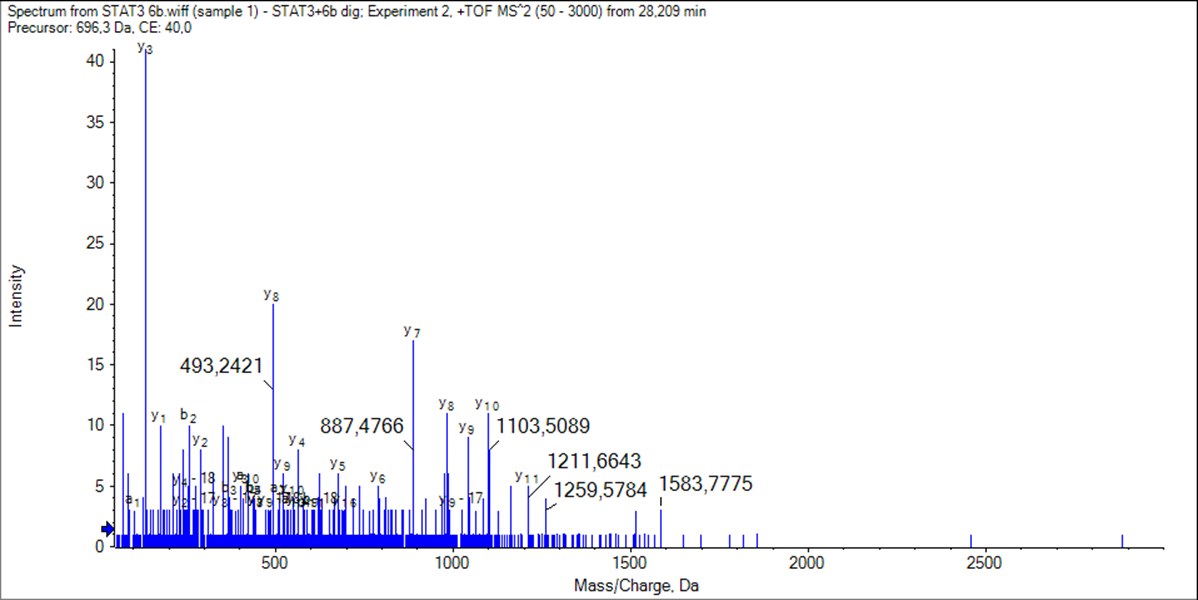

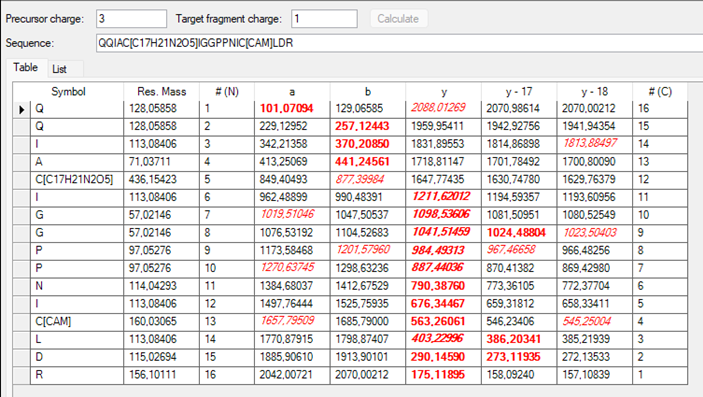


# **Figure S6**. Fluorescence polarization assay results. Nonlinear curve fitting was used ([Inhibitor] vs. response - Variable slope (four parameters)) with least squares fitting. IC_50_s are presented with 95% confidence intervals, measurements were done in 3 parallel biological replicas.

**SAR table**


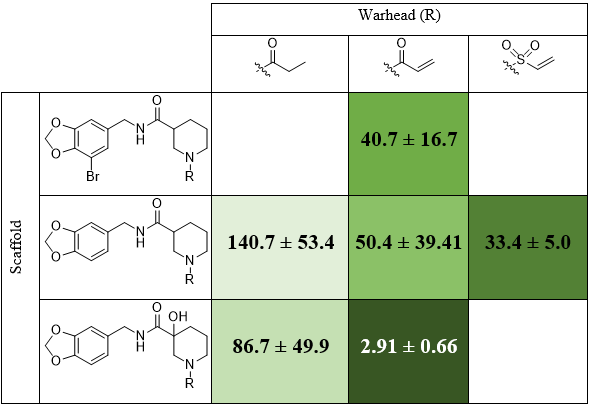


**IC_50_ titration curves**

**K2**

IC_50_: 40.7 ± 16.7 µM

R^2^: 0.9906


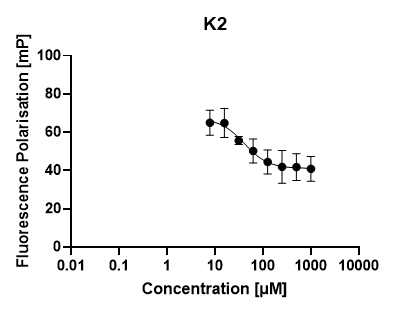


**K3**

IC_50_: 255.2 ± 124.1 µM

R^2^: 0.9925


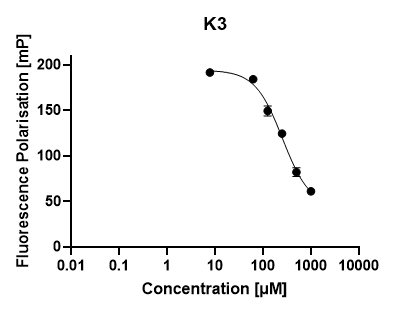


**K6**

IC_50_: 44.9 ± 22.7 µM

R^2^: 0.9886


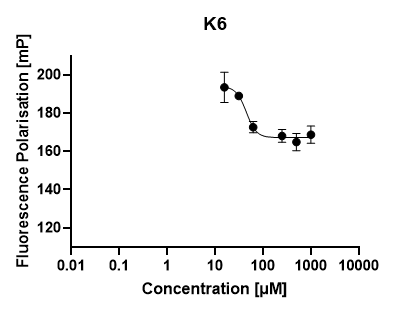


**K10**

IC_50_: 94.6 ± 32.5 µM

R^2^: 0.9873


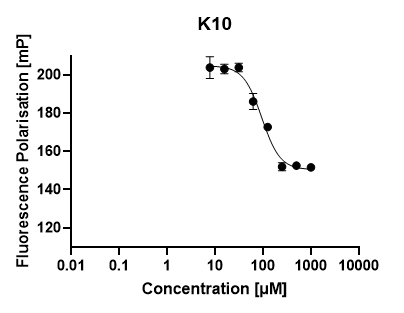


**6a**

IC_50_: 50.4 ± 39.41 µM

R^2^: 0.9733


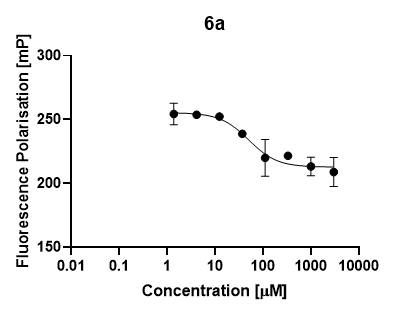


**6b**

IC_50_: 2.91 ± 0.66 µM

R^2^: 0.9975


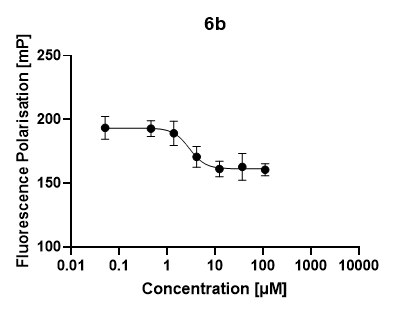


**8**

IC_50_: 33.4 ± 5.0 µM

R^2^: 0.9970


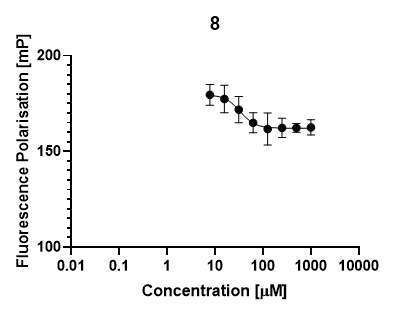


**10a**

IC_50_: 140.7 ± 53.4 µM

R^2^: 0.9688


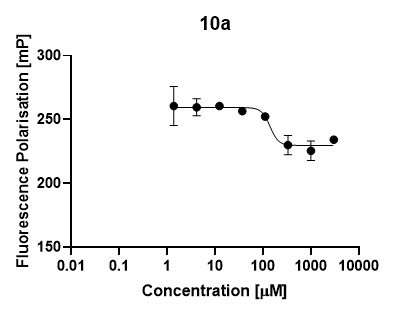


**10b**

IC_50_: 86.7 ± 49.9 µM

R^2^: 0.9975


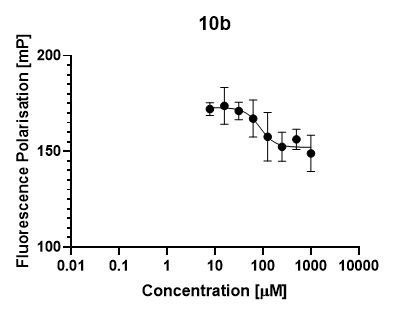

Supplement: Supplementary file 1 [file ml4c00622_si_001.docx]
